# New Derivatives of 1-(3-Methyl-1-Benzofuran-2-yl)Ethan-1-one: Synthesis and Preliminary Studies of Biological Activity

**DOI:** 10.3390/ijms25041999

**Published:** 2024-02-07

**Authors:** Mariola Napiórkowska, Pratheeba Kumaravel, Mithulya Amboo Mahentheran, Ewelina Kiernozek-Kalińska, Emilia Grosicka-Maciąg

**Affiliations:** 1 Chair and Department of Biochemistry, Medical University of Warsaw, 1 Banacha Str., 02-097 Warsaw, Poland; s079330@student.wum.edu.pl (P.K.); mithulya.amboo.5@gmail.com (M.A.M.); 2 Department of Immunology, Faculty of Biology, University of Warsaw, 1 Miecznikowa Str., 02-096 Warsaw, Poland; 3 Department of Biochemistry and Laboratory Diagnostic, Collegium Medicum Cardinal Stefan Wyszyński University, Kazimierza Wóycickiego 1 Str., 01-938 Warsaw, Poland; e.grosicka-maciag@uksw.edu.pl

**Keywords:** benzofurans, synthesis, cytotoxicity, apoptosis, anticancer, antibacterial

## Abstract

A set of nine derivatives, including five brominated compounds, was synthesized and the structures of these novel compounds were confirmed using ^1^H and ^13^C NMR as well as ESI MS spectra. These compounds were tested on four different cancer cell lines, chronic myelogenous leukemia (K562), prostate cancer (PC3), colon cancer (SW620), human kidney cancer (Caki 1), and on healthy human keratocytes (HaCaT). MTT results reveal that two newly developed derivatives (**6** and **8**) exhibit selective action towards K562 cells and no toxic effect in HaCat cells. The biological activity of these two most promising compounds was evaluated by trypan blue assay, reactive oxygen species generation, and IL-6 secretion. To investigate the proapoptotic activity of selected compounds, the two following types of tests were performed: Annexin V Apoptosis Detection Kit I and Caspase-Glo 3/7 assay. The studies of the mechanism showed that both compounds have pro-oxidative effects and increase reactive oxygen species in cancer cells, especially at 12 h incubation. Through the Caspase-Glo 3/7 assay, the proapoptotic properties of both compounds were confirmed. The Annexin V-FITC test revealed that compounds **6** and **8** induce apoptosis in K562 cells. Both compounds inhibit the release of proinflammatory interleukin 6 (IL-6) in K562 cells. Additionally, all compounds were screened for their antibacterial activities using standard and clinical strains. Within the studied group, compound **7** showed moderate activity towards Gram-positive strains in antimicrobial studies, with MIC values ranging from 16 to 64 µg/mL.

## 1. Introduction

Leukemias remain an unsolved problem in the clinical medicine and pharmacology of the 21st century. Although many clinically useful anticancer compounds are available on the pharmaceutical market, there are still obstacles in providing optimal therapies for patients. The main challenge in leukemia therapies is the shortage of drugs that specifically target cancer cells but that do not damage normal cells. In addition, a major problem is the development of multidrug resistance and the shortage of drugs effective against cancer-initiating stem cells, which may be the source of cancer recurrence [1,2].

Drugs used in the treatment of chronic myeloid leukemia (CML) and other leukemias include Imatinib (IM), bortezomib, and doxorubicin [3,4,5,6,7,8,9]. While therapies based on Imatinib effectively treat many patients, they still fall short in terms of eliminating the root cause of cancer—the cancerous stem cells. Moreover, during Imatinib therapy, the development of resistance to IM is observed, especially in the advanced stage of the disease [10,11,12,13]. 

The therapeutic value of bortezomib and doxorubicin is diminished due to a lack of selectivity towards cancer cells and these treatments induce strong side effects [8,9]. Fraczkowska et al. have proved that doxorubicin affects the mechanical properties of cellular structures via the reduction of cell stiffness. They have also showed that doxorubicin interacts with both proliferating cancer cells and normal healthy cells and that this can be further linked to the high toxicity of doxorubicin at its therapeutic concentrations [9]. Bortezomib has been successfully applied to the treatment of multiple myeloma and mantle cell non-Hodgkin’s lymphoma (NHL). However, it should be highlighted that, despite the successful use of bortezomib in myeloma and mantle cell lymphoma treatment, resistance to this drug remains a clinically significant problem. Results of clinical studies have revealed that resistance to bortezomib can be inherent or acquired [14]. Additionally gathered clinical observations indicate that bortezomib therapy is linked with some side effects, including peripheral neuropathy, orthostatic hypotension, pyrexia, cardiac and pulmonary disorders, gastrointestinal adverse events, myelosuppression, thrombocytopenia, asthenia, and pain [15,16]. Therefore, there is a great need to search for more effective chemotherapeutic compounds with high selectivity and minimal side effects on patients. 

In the field of medicinal chemistry, natural and synthetic benzofuran derivatives are well known for their wide therapeutic properties. These properties span various areas, including antiviral, antitumor, antihyperglycemic, analgesic, anti-inflammatory, antibacterial, and antifungal effects [17,18,19,20,21,22,23]. The multifaceted actions of benzofuran derivatives have positioned them as a subject of significant research, particularly in the realm of anticancer activity. Numerous active compounds with diverse structures have been identified, demonstrating cytotoxic activity against various cancer cell lines [24,25,26,27,28,29,30,31]. It has been shown that the anticancer effect of the abovementioned compounds results from, among other factors, the induction of apoptosis by caspase-dependent pathways in cancer cells or by the effective induction of cell death mediated by autophagy or by inhibition of tubulin polymerization. The other proposed mechanisms are the inhibition of angiogenesis, DNA synthesis, the induction of cell cycle arrest or the manipulation of ROS levels or IL-6 concentration levels [29,30,31].

Our team has dedicated several years to researching benzofuran derivatives, leading to the identification of bromo derivatives with potent anticancer activity. Through evaluation of the relationship between the structure of selected compounds and their activity, we have discovered that bromoalkyl and bromoacetyl derivatives of benzofurans exhibit the highest cytotoxicity. Our studies on the molecular mechanism of action have revealed that the most active benzofurans induce apoptosis in K562 and MOLT-4 leukemia cells [32,33,34,35]. Moreover, we have identified tubulin as the molecular target for the mentioned derivatives.

**Figure 1:**
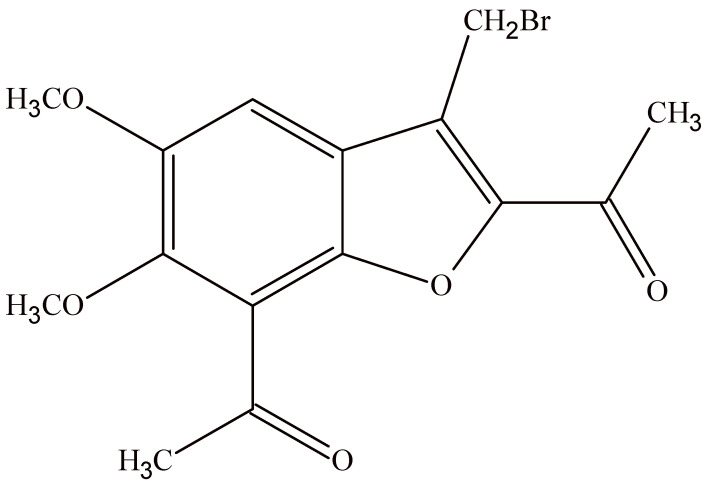
1,1′-[3-(bromomethyl)-5,6-dimethoxy-1-benzofuran-2,7-diyl]diethanone.

Among the compounds studied, 1,1′-[3-(bromomethyl)-5,6-dimethoxy-1-benzofuran-2,7-diyl]diethanone (Figure 1) emerged as the most promising anticancer agent. This derivative demonstrates selective toxicity against leukemic cell lines (K562, HL-60) with IC50 values of 5.0 and 0.1 mM, respectively, while exhibiting toxicity toward neither HeLa cancer cells nor non-cancer lines (HUVEC).

Building on these promising results, we used the mentioned derivative as the lead compound and synthesized nine new derivatives of benzofurans, including five bromo derivatives. The chemical modifications involved introducing substituents, –OCH_3_ and –OCH_2_CH_3_, at selected positions on the benzene ring. The type and position of these substituents influence the volume, lipophilicity, and activity of the new derivatives, providing valuable insights into the relationships between their structures and biological activity. Next, we evaluated their cytotoxicity against four cancer cell lines and studied the ability of the most active derivatives to induce apoptosis, ROS generation, and inhibit interleukin-6 release. Finally, the obtained results allowed us to analyze how modifications in the derivatives and the position of substituents on the benzene ring impact their cytotoxic and biological activity compared with the lead compound.

## 2. Results and Discussion

### 2.1. Chemistry

**Scheme 1:**
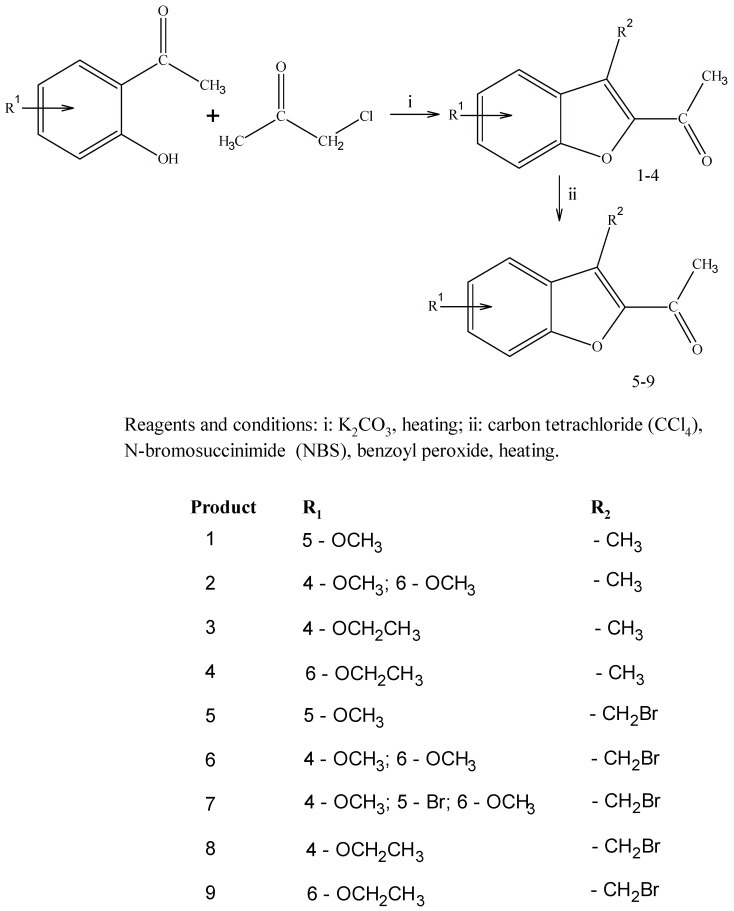
Synthesis of selected 1-(3-methyl-1-benzofuran-2-yl)ethanone derivatives **1**–**9**.

The main goal of our synthetic studies was to obtain a small library of novel derivatives of selected benzofurans. The route of preparation of described compounds is presented in Scheme 1. The starting benzofurans were obtained in the reaction between an appropriate substituted or unsubstituted o-hydroxyacetophenone and chloroacetone. Next, obtained compounds were brominated by using NBS (N-bromosuccinimide) in CCl_4_ (carbon tetrachloride). As a result, the bromomethyl derivatives were obtained for all starting benzofurans. An additional product containing two bromine atoms, in the methyl group and in the benzene ring, was isolated in the case of 1-(4,6-dimethoxy-3-methyl-1-benzofuran-2-yl)ethanone. We assumed that the presence of the –OCH_3_ groups of the benzene rings assisted in the electrophilic substitution in the ortho position of the benzene ring. 

All of the obtained derivatives were purified by silica gel column chromatography and their structures were confirmed by ^1^H nuclear magnetic resonance (NMR), ^13^C NMR, and mass spectrometry (MS). The obtained compounds were tested for their cytotoxic properties on human cancer cells and healthy cells, and they were screened for antibacterial activities using standard and clinical strains. The NMR spectra of the investigated compounds are provided as Supplementary Materials.

### 2.2. Biological Evaluation 

#### 2.2.1. MTT Cytotoxicity Studies

The results of the following MTT screening study of nine novel benzofurans derivatives presents cytotoxic activity in the four following cancer cell lines: chronic myelogenous leukemia cell (K562), human prostate cancer cell (PC3), colorectal cancer cell (SW620), and human renal cell carcinoma cell (Caki-1). Activity was also present in normal human keratinocytes (HaCaT). The cell viability was analyzed at the following six concentrations of compounds: 5, 10, 20, 40, 60 and 100 µM after 72 h incubation.

Based on the obtained results we calculated IC_50_ (µM) values (the concentration of the compound that corresponds to a 50% growth inhibition of cell lines as compared with the control) and present them in Table 1. Additionally, to determine the safety profile of the tested compounds, the therapeutic index (TI) (the ratio of IC_50_ value in HaCaT to cancer cells K562) was calculated. Firstly, the obtained results clearly show that all examined benzofuran derivatives were not toxic towards kidney cancer cells, Cacki-1 (IC_50_ > 100 µM). However, the derivatives exhibited different cytotoxic potentials in other cell lines used in our study. It should be stressed that the introduction of bromine into benzofuran molecules increases their cytotoxic potential in both normal and cancer cells (except for the previously mentioned Cacki-1 cells). Analysis of the obtained results allowed us to select compounds with low harmful effects on normal cells. Compounds **1**–**4** exhibited lower cytotoxic potential in HaCat cells compared with the brominated derivatives **5**–**9**, with IC_50_ values that ranged from 50–100 µM. Unfortunately, there were no cytotoxicity activities induced towards K562, PC3 or SW620 cells (IC_50_ > 30 µM; Table 1). Thus, we decided to exclude these from our further studies. Compounds **5**–**9**, which belong to brominated molecules, exhibit stronger cytotoxic effects both in normal and cancer cells. Compound **5** unveiled similar harmful effects to both healthy and cancer cells (IC_50_ = 14.6 ± 3.5 µM) and hence further analysis was not conducted. Compound **6** showed selective strong toxic effects in K562 (IC_50_ = 3.83 ± 0.6 µM) and moderate toxic effects in HaCaT (12.44 ± 1.27 µM), with TI = 3.248. Because compound **7** was extremely toxic towards HaCaT cells it was also excluded from our study (Table 1). Compound **8** acts the same as compound **6,** demonstrating a high selective toxic potential in K562 cells (IC_50_ = 2.59 ± 0.88 µM) and a moderate toxic effect in HaCaT cells (23.57 ± 10.7 µM), with TI = 9.100. Compound **9**, in a similar fashion to compound **5**, failed to expose selective toxicity against cancer cells (Table 1). The goal of conducting the MTT assay in this research was to select benzofuran derivatives, which manage to reduce viability of cancer cells while exposing mild effects on normal healthy cells. Among all nine of the examined benzofurans, we decided to select two compounds, **6** and **8.** It should be stressed that both compounds exhibit moderate values of therapeutic index (TI), 3.248 for **6** and 9.100 for **8**, but they are still significantly less toxic compared with commonly used chemotherapeutics, including bortezomib or doxorubicin. 

#### 2.2.2. Antiproliferative Activity

The MTT assay assesses the viable cell number based on mitochondrial enzyme activity. Thus, the results of an MTT analysis do not allow us to estimate the total cell number. In the next step of our studies, we analyzed the total cell number and viability after exposure to the selected compounds at IC_50_ concentration using trypan blue exclusion assay. K562 cells were incubated with tested compounds over 72 h. Compounds **6** and **8** did not significantly affect the cell viability compared with the untreated K562 cells (Table 2). To assess the antiproliferative properties of newly synthesized derivatives, we analyzed the total cell number. Incubation with compound **8** did not affect the total cell number compared to the control. On the other hand, compound **6** exhibited slight antiproliferative property and reduced cell number by 13%. The observed difference in activity of both tested compounds might result from different patterns of substituents in the benzene ring (Table 2). Compound **8** possesses –OCH_2_CH_3_ attached to carbon 4 in the benzene ring whereas compound **6** has two substituents but both consist of one carbon atom. It seems that both the size and pattern of substituents in the benzene ring influence the polarity of tested derivatives. We can thus conclude that the structure of compound **6** facilitates its transport through phospholipid cellular membrane and other intracellular membranes. Based on these findings we suppose that biological action, including the antiproliferative effect of compound **6**, might be observed faster and evidently. The parallel experiment was conducted with HaCaT cells. The obtained results reveal that both tested derivatives exhibit mild cytotoxic activity in normal cells, but observed changes were not significantly important. However, it should be highlighted that HaCaT cells viability in both examples were higher than 80%. The trypan blue test rapidly assesses cell viability indirectly from cell membrane integrity, but some publications have indicated weaknesses of this viability test [36,37,38]. Strober [38] suggests that it is possible that a cell’s viability might be affected (as estimated by capacity to grow or function) even when its membrane integrity is (at least transiently) maintained. On the other hand, cell membrane integrity may be affected even while the cell might still be able to repair itself and become fully viable. The observed slightly higher cytotoxic effect in HaCaT cells compared with K562 seems to be a normal cellular response to potential toxic factors. Cancer cells are equipped with perfectly developed defense mechanisms which allow them to survive in different environments, including in the presence of potential anticancer compounds, whereas normal cells have to create, adapt and activate the molecular mechanisms which allow them to survive in the presence of unfavorable environmental conditions. 

#### 2.2.3. Reactive Oxygen Species (ROS) Generation

**Figure 2:**
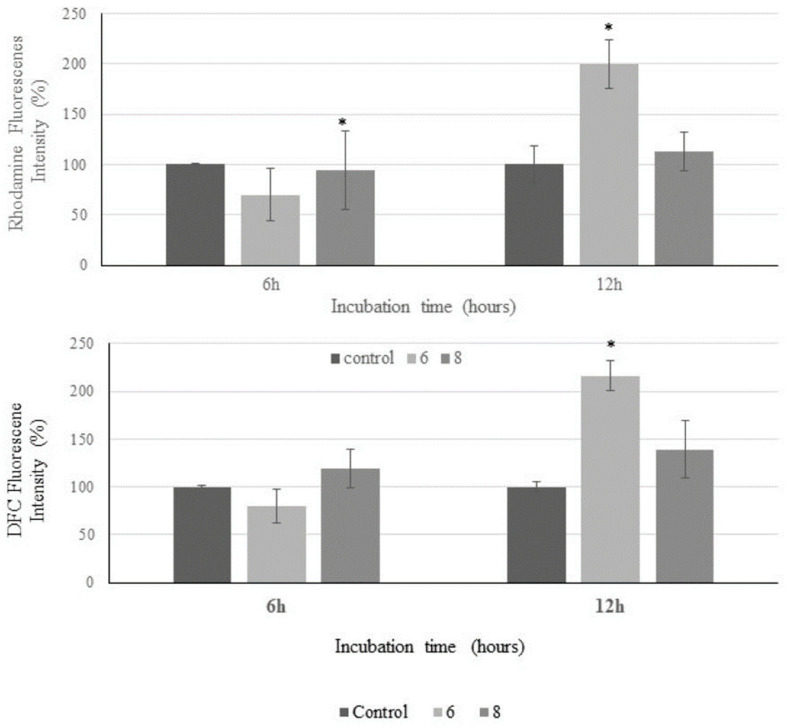
The effect of the benzofuran derivatives on ROS generation in K562 cancer cell lines treated with compounds **6** and **8** for 6 h and 12 h, respectively, measured by the spectrofluorimetric method. Data are expressed as the mean ± SD from three independent experiments performed in triplicate. * *p* < 0.05 as compared to the control cells.

Literature dates indicate that active benzofurans and their derivatives exhibit both oxidant and antioxidant properties [31,39,40,41]. In the present study ROS generation examination was performed after 6 and 12 h exposure to tested derivatives at IC_50_ concentration. The analysis is based on ROS-dependent oxidation of the compounds to fluorescent rhodamine 123 and dichlorofluorescein (DCF), respectively. In K562 cells incubated with either dihydrorhodamine (123 DHR123) or 2′,7′-dichlorodihydrofluorescein diacetate (DCF-DA), a statistically significant increase of fluorescence intensity was observed after 12 h incubation with compound **6** and **8** (Figure 2). Observed changes in fluorescence intensity indicate that both compounds increase the concentration of H_2_O_2_, which is responsible for the oxidation of DHR123 and DCF–DA. However, both compounds differ in their oxidative potential. We noticed that compound **6** exhibits the stronger pro-oxidative effect in K562 cells as compared with compound **8.** It induces a two-fold increase in fluorescence intensity compared with untreated cells. Observed changes in the oxidant properties of tested benzofurans confirmed our previous findings that even small differences in the pattern and types of substituents in aromatic rings might affect biological activity. In our research we used two dyes, which allowed us to detect H_2_O_2_. Hydrogen peroxide is the product of reaction catalyzed by superoxide dismutase (SOD). In this reaction, superoxide O_2_^−^ is converted into O_2_ and H_2_O_2_. An observed higher concentration of hydrogen peroxide after benzofurans exposure indicate that their oxidative action in leukemia cells might be associated with superoxide generation. Our results are in agreement with studies presented by Zhou et al., who have revealed that chronic lymphocytic leukemia cells (CLL) obtained from patients exhibit higher ROS production compared with normal lymphocytes [42]. Additionally, the work of Jitschin et al. indicates that CLL patients have displayed a metabolic condition known as oxidative stress and that this is linked to alterations in their lymphoid compartment [43]. Additionally, their research showed that mitochondrial metabolism is a main source of ROS. It must be highlighted that ROS exhibit activity is a “double-edged sword” in cancer cells. When ROS are present at low-to-moderate concentrations, they act as signaling transducers to activate cancer cell proliferation, migration, invasion, angiogenesis, and drug resistance [44,45,46,47]. Simultaneously the high level of ROS might be harmful to cancer cells and ultimately lead to cell death [48]. Thus, it seems reasonable that modulation of ROS levels and the antioxidant barrier in cancer cells might be key points for the development of effective anticancer therapy.

#### 2.2.4. Inhibition of IL-6 Release

Among many soluble factors present in the plasma and tissue microenvironment in patients with chronic lymphatic leukemia recent studies confirmed a presence of few types of interleukin (IL) including 4, 6 and 10. Additionally, research indicates that they may play an important function in supporting cell survival [49,50,51,52]. IL-6 is a member of a big family of cytokines and is predominantly excreted by T cells and macrophages to stimulate immune response during infection [53]. Studies have shown that elevated levels of serum IL-6 is positively correlated with worse clinical outcomes in patients with different types of cancer, including leukemia [54,55]. IL-6 also plays a key role in the chemoresistance of malignancies [56,57,58]. Based on these findings we checked the effect of our new benzofuran derivatives on IL-6 secretion by K562 cells. We analyzed the concentrations of IL-6 in cell culture medium after 72 h exposure to compound **6** or **8**. The obtained results show that both compounds significantly reduced the concentration of IL6 in the tested medium. The stronger effect was observed after treatment with compound **6**. This reduced the level of IL-6 by 50%, whereas compound **8** reduced it by 40% (Table 3). These results confirm our earlier observations regarding the biological properties of the tested compounds. We showed that compound **6** has a stronger pro-oxidative effect in K562 cells. Therefore, based on the obtained results we might hypothesize that the observed increase in the concentration of free radicals might affect the level of released interleukin 6. This hypothesis is surprising, because studies clearly indicate that synthesis and release of IL-6 occurs in response to the stimulation of different factors including ROS, TNF or LPS. On the other hand, K562 cells like other erythroid and megakaryocytic cells release IL-6 in a constitutive way [59]. Additionally, studies conducted by Schuringa et al. have revealed that 25% AML patients exhibit constitutive Stat3 activation and secret high levels of IL-6. Blocking the action of secreted IL-6 by treatment with anti-IL-6 leads to a loss of constitutive Stat3 phosphorylation and normal IL-6-induced Stat3 activation patterns. These suggest that the autocrine and paracrine secretion of IL-6 by AML cells causes the constitutive activation of Stat3, which may have important consequences for the growth and survival characteristics of AML cells [60]. Interesting results were obtained also by Liang et al. They demonstrated that STAT3 activation inhibited ROS overload. Furthermore, decreased concentration of ROS may influence transcription factor activation and promote cell proliferation and differentiation [61]. Based on these findings we suppose that observed decreased IL-6 secretion might be the result of pro-oxidative activity of both compounds, as a consequence of the modulation of the STAT3/ROS axis in K562 cells. 

#### 2.2.5. Activation of Apoptosis

**Figure 3:**
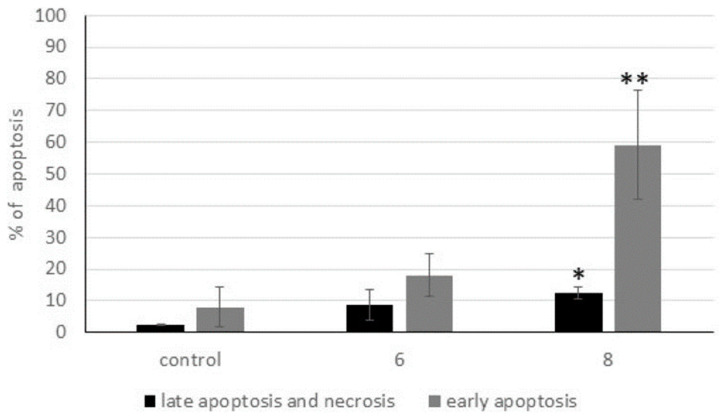
The effect of compounds 6 and 8 on early and late apoptosis or necrosis in K562 cells. * *p* ≤ 0.05, ** *p* ≤ 0.01 as compared with the control cells.

**Figure 4:**
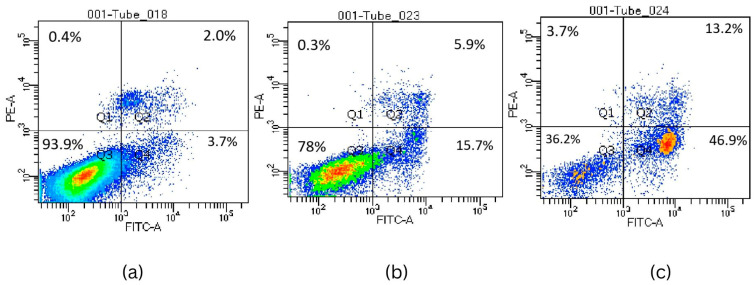
Representative results (%) as dot plots from apoptosis analysis of K562 after treatment with selected compounds tested with flow cytometry. Q1, necrotic cells; Q2, late apoptotic cells; Q3, live cells; Q4, early apoptotic cells; (**a**) control cells, (**b**) cell treated with compound 6, and (**c**) cells treated with compound **8**.

**Figure 5:**
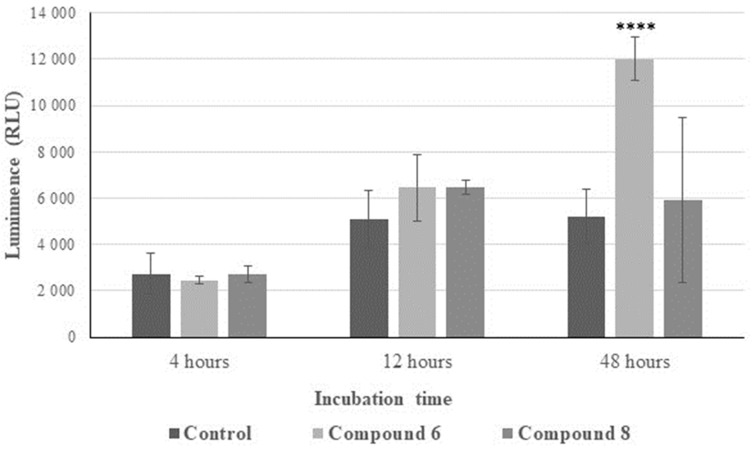
The effect of selected compounds on caspase 3/caspase 7 activity. Cells were incubated for 12, 24, or 48 h with the tested compounds used at their IC50 concentrations. The caspase levels were analyzed using Caspases-Glo3/7 assay system after cell incubation at 4, 12 and 48 h. Data are expressed as mean ± SD from three independent experiments performed in triplicate. **** *p* ≤ 0.001 as compared to the control cells.

ROS may play a different function in different stages of cancer. ROS might enhance cell growth and proliferation through the induction of various cell signaling pathways involving nuclear factor-kappa B (NF-κB) and STAT3, hypoxia-inducible factor 1α (HIF1α), kinases, growth factors, cytokines and many other proteins. On the other hand, the accumulation of ROS can induce cell damage and promote apoptosis. It has also been confirmed that elevated ROS concentration may cause mitochondrial membrane depolarization and facilitate increased cellular pro-apoptotic molecules releasing [62,63,64]. In the present study we showed that compounds **6** and **8** increase the level of ROS in K562 cells in a time-dependent manner. As has been previously reported, lead compound 1,1′-[3-(bromomethyl)-5,6-dimethoxy-1-benzofuran-2,7-diyl] induces apoptosis in human leukemia cells [34,35]. Therefore, we can suppose that both new derivatives might possess apoptotic potential. To confirm the ability of our tested benzofurans to induce apoptosis, we analyzed phosphatidylserine (PS) distribution in K562 cells exposed to compound **6** or **8**. We used the Annexin V-FITC test, which allows us to detect the early structural changes in cellular membrane which occur during apoptosis. The results of flow cytometry analysis showed that compounds **6** and **8** induced apoptosis (early and late stages) in 21.6% and 60.1% of K562 cells, respectively (Figure 3 and Figure 4). In the control sample almost 94% of cells were neither apoptotic nor necrotic. The observed stronger signal for Annexin in cells exposed to compound **8** may indicate that the apoptosis process is more advanced than in cells exposed to compound **6**. Compound **8** also revealed the stronger cytotoxic potential in MTT analysis. As has been previously written, the MTT test assesses the toxicity of tested compounds based on the activity of mitochondrial enzymes. Based on these findings we suppose that compound **8** might possess the stronger effect on mitochondrial metabolism. Our results are in agreement with studies conducted by Liu et al. and Su et al. They report that the benzofuran derivatives ACDB and BL-038 induce apoptosis of human chandrosarcoma cells through ROS generation, mitochondria and endoplasmic reticulum dysfunction. BL-038 increases ROS production and dysregulates mitochondria membrane potential. This causes cytochrome C to be released from mitochondria and caspase activation. Similar effects have been observed for 2-amino-3-(2-chlorophenyl)-6-(4-dimethylaminophenyl) benzofuran-4-yl acetate (ACDB) [65,66]. It is well documented that disruption of the mitochondrial membrane as a result of, for example, the action of free radicals causes the release of cytochrome C. In the next step, cytochrome C binds to Apaf-1 (apoptotic protease activating factor 1) and it becomes the main component of apoptosome [67]. The binding of cytochrome C with Apaf-1 is a critical step in apoptosome formation because it is responsible for the induction of intrinsic cell death pathway triggered by caspase activation. The observed increased concentration of hydrogen peroxide in cells exposed to both tested compounds might be a key factor for the molecular mechanism of apoptosis. Hydrogen peroxide can affect the redox state of cytochrome C. In addition to the effect of hydrogen peroxide on the iron atom in the cytochrome, it can also affect the activation of caspases. In order to estimate the effect of K562 exposure on the tested compounds as well as to confirm apoptotic properties of new benzofurans, we analyzed the effect of the compounds caspases 3 and 7 activities. We checked the activity of both caspases after 4, 12 and 48 h exposure to compound **6** or **8** (Figure 5). Obtained results show that 4 h incubation did not affect the activity of caspases 3 and 7, after 12 h exposure we observed a slight increase of the activities of both caspases. Compound **6** increased activity by 26%, whereas compound **8** increased activity by 27%. We observed significant changes of activity after 48 h exposure. Compound **6** exhibited very strong proapoptotic activity, we noted a 2.31-fold increase of activity of caspases 3 and 7. On the other hand, compound **8** increased the activity of both caspases only by 13%. The results of both analyses reveal that the two tested compounds exhibit different potentials in apoptosis induction in K562 cells. As has been mentioned previously, we supposed that compound **8** might induce apoptosis via mitochondrial pathway. On the other hand, we observed the stronger effect of compound **6** on both executive caspases 3 and 7. The caspase cascade responsible for executing cell death following cytochrome C release is well documented. Available research indicates, first of all, the key role of caspases 3 and 7 as mediators in the apoptosis through the intrinsic (mitochondrial) cell death pathway. Furthermore, the work of Brentall et al. has demonstrated that three executive caspases, 3, 7 and 9, have distinct roles during intrinsic apoptosis. They have shown that caspase 9 is responsible for mitochondrial morphological changes and ROS production by cleaving and activating Bid into tBid. Following caspase 9 activation, caspase 3 diminishes ROS generation and is essential for the efficient execution of apoptosis, whereas effector caspase 7 is needed for apoptotic cell detachment [68,69]. The complicated intracellular apoptotic signals are finally transduced into a phospholipid pattern in cellular membrane and phosphatidylserine (PS) translocation. Based on these data we might conclude that both tested derivatives induce apoptosis through intrinsic pathways associated with mitochondria and ROS generation. However, the obtained results also show that both the type and arrangement of substituents in the aromatic ring has an impact on the rate of induction and the course of the apoptosis process.

### 2.3. Antibacterial Studies

All of the obtained derivatives (**1**–**9**) were tested in vitro against a number of bacteria, including representative standard Gram-positive and Gram-negative rods. Compounds were screened for their minimal inhibitory concentrations (MICs) [70,71]. 

Of the compounds tested, only compound **7** showed moderate antimicrobial activity with MIC values ranging from 16 to 64 µg/mL (Table 4). This compound exhibited growth-inhibitory properties towards Gram-positive strains, with the exception of the *S. aureus* NCTC 4163 strain. It should be emphasized that the activity of this derivative seems to be determined by the presence of bromine substituted in the aromatic ring. This is confirmed by the comparison of the activity of this compound with compound **6**, which differs only in the substitution in the aromatic ring and which did not show antimicrobial properties against the studied strains. The remaining tested compounds also do not have bromine in the aromatic ring and have not shown antimicrobial activity against tested strains. Inactive compounds were not included in the table.

Next, compound **7** was tested against clinical strains. Unfortunately, the tested derivative did not show antimicrobial activity against the employed clinical strains (MIC > 250 ug/mL). The results are not shown.

## 3. Material and Methods

### 3.1. Chemistry

All commercial reagents, solvents and chemicals used in the present studies were purchased from Aldrich Chemical Company (Saint Louis, MO, USA) and Alfa Aesar (Haverhill, MA, USA) and used without purification. Melting points were measured on an Elektrothermal 9100 (Elektrothermal, Kraków, Poland) capillary apparatus and were uncorrected. The 1H NMR and 13C NMR spectra were recorded on a Bruker BioSpin GmbH spectrometer (Bruker, Billerica, MA, USA) operating at 300 MHz (^1^H NMR) and 75.49 MHz (^13^C NMR) in DMSO-d_6_ or CDCl_3_, respectively. Chemical shifts (δ) are expressed in parts per million relative to tetramethylsilane (TMS), which was used as the internal reference. Coupling constants (*J*) values are given in hertz (Hz) and spin multiples are given as s (singlet), d (doublet), t (triplet), m (multiplet).

The mass spectra (MS) measurements were carried out on an LCT Micromass TOF HiRes apparatus (SpectraLab Scientific Inc., Markham, ON, Canada) and were obtained in the positive ion mode. Column chromatography was performed on Merck Kieselgel (Darmstadt, Germany) 0.05–0.2 mm reinst (70–325 mesh ASTM) silica gel using hexane as an eluent or eluent system with hexane: ethyl-acetate (100:5 *v*/*v*). Progress of the reactions described in the experimental section was monitored by TLC on silica gel (plates with fluorescent indicator 254 nm, layer thickness 0.2 mm, Kieselgel G. Merck), using chloroform: methanol as an eluent system at the *v*/*v* ratio of 9.8:0.2 or 9.5:0.5.

### 3.2. Synthetic Procedures

#### 3.2.1. Synthesis of Starting Benzofurans 1–4

The starting benzofurans were prepared according to the method described in the literature [72].

Thus, to a solution of appropriately substituted or unsubstituted o-hydroxyacetophenone (16.6 mmol) and chloroacetone (16.6 mmol, 1.32 mL) in acetonitrile (30 mL) was added K_2_CO_3_ (33.2 mmol, 4.6 g). The obtained mixture was heated at 80 °C and stirred for 48–96 h. When the reaction was completed, the mixture was diluted with H_2_O (20 mL) and extracted with Et_2_O (3 × 30 mL). The combined organic extracts were washed with NaOH (0.5 mol/L in H_2_O 1 × 50 mL) and saturated NaCl (1 × 50 mL), dried with Na_2_SO_4_ and concentrated under reduced pressure. The obtained residue was purified by a silica gel column chromatography using hexane as an eluent or hexane: ethyl-acetate (100:5 *v*/*v*) as an eluent system.

##### 1-(5-Methoxy-3-Methyl-1-Benzofuran-2-yl)Ethanone (**1**)

M.W. = 204.2218; C_12_H_12_O_3_; yield: 60%; white powder, m.p. 93–96 °C; ^1^H NMR (300 MHz, CDCl_3_, δ/ppm): 2.57 (3H, *s*, -CH_3_), 2.59 (3H, *s*, -COCH_3_), 3.87 (3H, *s*, -OCH_3_), 7.00 (1H, *m*, Ar-H), 7.07 (1H, *dd*, *J*_1_ = *J*_2_ = 2.4 Hz, Ar-H), 7.40 (1H, *m*, Ar-H); ^13^C NMR (75.49 MHz, CDCl_3_) δ: 9.56, 27.70, 55.87, 102.22, 112.83, 118.26, 124.10, 129.80, 148.98, 149.04, 156.23, 191.26; HRMS (*m*/*z*): calculated value for C_12_H_12_O_3_ [M + Na]: 227.0684; found: 227.0674.

##### 1-(4,6-Dimethoxy-3-Methyl-1-Benzofuran-2-yl)Ethanone (**2**)

M.W. = 234.2478; C_13_H_14_O_4_; yield: 30%; white powder, m.p. 115–118 °C; ^1^H NMR (300 MHz, CDCl_3_, δ/ppm): 1.79 (3H, *s*, -CH_3_), 2.35 (3H, *s*, -COCH_3_), 3.87 (3H, *s*, -OCH_3_), 4.00 (3H, *s*, -OCH_3_), 6.58 (1H, *m*, Ar-H), 6.94 (1H, *m*, Ar-H); ^13^C NMR (75.49 MHz, CDCl_3_) δ: 10.77, 28.04, 57.16, 61.18, 108.64, 114.82, 125.48, 128.83, 133.86, 147.25, 148.57, 151.38, 190.95; HRMS (*m*/*z*): calculated value for C_13_H_14_O_4_ [M + Na]: 257.0790; found: 257.0779.

##### 1-(4-Ethoxy-3-Methyl-1-Benzofuran-2-yl)Ethanone (**3**)

M.W. = 218.2484; C_13_H_14_O_3_; yield: 60%; white powder, m.p. 60–65 °C; ^1^H NMR (300 MHz, CDCl_3_, δ/ppm): 1.49 (3H, *t*, *J* = 7.05 Hz, -CH_2_-CH_3_), 2.57 (3H, *s*, -CH_3_), 2.76 (3H, *s*, -COCH_3_), 4,14 (2H, *q*, *J* = 6.9 Hz, -CH_2_-CH_3_), 6.58 (1H, *m*, Ar-H), 7.04 (1H, *m*, Ar-H), 7.32 (1H, *m*, Ar-H); ^13^C NMR (75.49 MHz, CDCl_3_) δ: 11.34, 14.71, 27.92, 63.86, 103.81, 104.61, 119.16, 125.60, 129.01, 147.15, 155.35, 156.33, 191.10; HRMS (*m*/*z*): calculated value for C_13_H_14_O_3_ [M + Na]: 241.0841; found: 241.0836.

##### 1-(6-Ethoxy-3-Methyl-1-Benzofuran-2-yl)Ethanone (**4**)

M.W. = 218.2484; C_13_H_14_O_3_; yield: 70%; white powder, m.p. 86–90 °C; ^1^H NMR (300 MHz, CDCl_3_, δ/ppm): 1.46 (3H, *t*, *J* = 7.05 Hz, -CH_2_-CH_3_), 2.56 (6H, *m*, -CH_3_, -COCH_3_), 4,07 (2H, *q*, *J* = 6.9 Hz, -CH_2_-CH_3_), 6.93 (2H, *m*, Ar-H), 7.49 (1H, *m*, Ar-H); ^13^C NMR (75.49 MHz, CDCl_3_) δ: 9.59, 14.67, 27.61, 63.95, 95.85, 113.86, 121.77, 122.65, 124.80, 148.05, 155.36, 160.37, 190.46; HRMS (*m*/*z*): calculated value for C_13_H_14_O_3_ [M + Na]: 241.0841; found: 241.0849.

#### 3.2.2. Procedure for Bromination by Using N-Bromosuccinimide (NBS)

An appropriate benzofuran (10 mmol) was dissolved in dry carbon tetrachloride (50 mL) before N-bromosuccinimide (NBS) (10 mmol, 1.78 g) and the catalytic amount of benzoyl peroxide (1 mmol, 0.242 g) were added. The reaction mixture was refluxed for 24–48 h. When the reaction was complete (TLC monitoring), the mixture was filtered and the solvent was removed under reduced pressure. Silica gel column chromatography purified the residue (eluent: hexane or hexane: ethyl-acetate; 100:5 *v*/*v*).

##### 1-[3-(Bromomethyl)-5-Methoxy-1-Benzofuran-2-yl]Ethanone (**5**)

M.W. = 283.11794; C_12_H_11_BrO_3_; yield: 30%; white powder, m.p. 103–106 °C; ^1^H NMR (300 MHz, CDCl_3_, δ/ppm): 2.62 (3H, *s*, -COCH_3_), 3.89 (3H, *s*, -OCH_3_), 5.03 (2H, *s*, -CH_2_Br), 7.14 (2H, *m*, Ar-H), 7.44 (1H, *m*, Ar-H); ^13^C NMR (75.49 MHz, CDCl_3_) δ: 21.16, 27.62, 55.90, 102.18, 113.13, 118.97, 123.33, 127.56, 148.13, 149.05, 156.59, 191.27; HRMS (*m*/*z*): calculated value for C_12_H_11_BrO_3_ [M + Na]: 306.9770; found: 306.9783.

##### 1-[3-(Bromomethyl)-4,6-Dimethoxy-1-Benzofuran-2-yl]Ethanone (**6**)

M.W. = 313.1439; C_13_H_13_BrO_4_; yield: 30%; red powder, m.p. 132–136 °C; ^1^H NMR (300 MHz, CDCl_3_, δ/ppm): 2.58 (3H, *s*, -COCH_3_), 3.86 (3H, *s*, -OCH_3_), 3.94 (3H, *s*, -OCH_3_), 5.01 (2H, *s*, -CH_2_Br), 6.33 (1H, *d*, *J* = 1.8 Hz, Ar-H), 6.59 (1H, *d*, *J* = 1.8 Hz, Ar-H); ^13^C NMR (75.49 MHz, CDCl_3_) δ: 22.59, 27.55, 55.80, 55.83, 87.75, 95.75, 110.93, 125.16, 146.68, 156.23, 156.30, 162.64, 190.12; HRMS (*m*/*z*): calculated value for C_13_H_13_BrO_4_ [M + Na]:336.9876; found: 336.9860.

##### 1-[5-Bromo-3-(Bromomethyl)-4,6-Dimethoxy-1-Benzofuran-2-yl]Ethanone (**7**)

M.W. = 392,03998; C_13_H_12_Br_2_O_4_; yield: 30%; red powder, m.p. 165–171 °C; ^1^H NMR (300 MHz, CDCl_3_, δ/ppm): 2.64 (3H, *s*, -COCH_3_), 4.00 (3H, *s*, -OCH_3_), 4.01 (3H, *s*, -OCH_3_), 5.08 (2H, *s*, -CH_2_Br), 6.43 (1H, *s*, Ar-H); ^13^C NMR (75.49 MHz, CDCl_3_) δ: 22.59, 27.65, 56.06, 57.19, 84.79, 92.35, 111.64, 125.39, 146.98, 153.00, 155.67, 157.94, 190.12; HRMS (*m*/*z*): calculated value for C_13_H_12_Br_2_O_4_ [M + Na]: 414.8980; found: 414.9003.

##### 1-[3-(Bromomethyl)-4-Ethoxy-1-Benzofuran-2-yl]Ethanone (**8**)

M.W. = 297.1445; C_13_H_13_BrO_3_; yield: 60%; white powder, m.p. 108–113 °C; ^1^H NMR (300 MHz, CDCl_3_, δ/ppm): 1.55 (3H, *t*, *J* = 7.05 Hz, -CH_2_-CH_3_), 2.62 (3H, *s*, -COCH_3_), 4,21 (2H, *q*, *J* = 6.9 Hz, -CH_2_-CH_3_), 5.17 (2H, *s*, -CH_2_Br), 6.66 (1H, *m*, Ar-H), 7.10 (1H, *m*, Ar-H), 7.38 (1H, *m*, Ar-H); ^13^C NMR (75.49 MHz, CDCl_3_) δ: 14.69, 22.59, 27.78, 64.22, 104.72, 104.81, 116.84, 124.75, 129.64, 146.90, 155.21, 155.61, 190.93; HRMS (*m*/*z*): calculated value for C_13_H_13_BrO_3_ [M + Na]: 320.9926; found: 320.9920.

##### 1-[3-(Bromomethyl)-6-Ethoxy-1-Benzofuran-2-yl]Ethanone (**9**)

M.W. = 297.1445; C_13_H_13_BrO_3_; yield: 60%; yellow powder, m.p. 98–105 °C; ^1^H NMR (300 MHz, CDCl_3_, δ/ppm): 1.36 (3H, *t*, *J* = 7.05 Hz, -CH_2_-CH_3_), 2.56 (6H, *m*, -COCH_3_), 4,12 (2H, *q*, *J* = 6.9 Hz, -CH_2_-CH_3_), 7.03 (1H, *dd*, *J*_1_ = *J*_2_ = 2.1 Hz, Ar-H), 7.29 (1H, *d*, *J* = 2.1 Hz, Ar-H) 7.76 (1H, *m*, Ar-H); ^13^C NMR (75.49 MHz, CDCl_3_) δ: 14.43, 22.06, 27.58, 63.88, 96.28, 114.58, 119.52, 122.36, 123.68, 146.77, 154.85, 160.31, 190.04; HRMS (*m*/*z*): calculated value for C_13_H_13_BrO_3_ [M + Na]: 320.9926; found: 320.9917.

### 3.3. Biological Studies

#### 3.3.1. MTT Cytotoxicity Studies

The cytotoxicity was evaluated by MTT reduction assay, which measures mitochondrial respiratory function. The test was performed based on the method described by Präbst et al. [73]. Cells (1 × 10^4^/well), in logarithmic phase, were seeded into 96-well plates in appropriate medium. After 24 h, cells were exposed to test compounds. The stock solution for each tested compound (freshly prepared in DMSO) was added to the cells to obtain final concentrations of 0.5, 1, 2, 6; 8 and 10 × 10^−2^ mM. The final concentration of DMSO did not exceed 0.1%. After 72 h, the medium with the compounds was completely removed and MTT solution (0.5 mg/mL) in fresh medium without serum was added to each well. Plates with cells were incubated for 4 h at 37 °C in the dark. Then, MTT solution was removed and replenished with 0.1 mL DMSO: isopropanol (1:1) mixture to dissolve the generated formazan crystals. The optical density was measured with a MULTISKAN GO (ThermoScientific) microplate reader at 570 nm. Replicates of three wells for each dosage, including vehicle control, were analyzed for each experiment. The experiments were conducted in triplicate. Control cells which were only treated with respective medium were used as the 100% viability value. The IC_50_ values (the concentration of a test compound required to reduce cell survival to 50% of the control) was estimated using GraphPad Prism Version 8.0.1 based on MTT results. Additionally, to determine the safety profile of the test compounds, therapeutic indexes (TI) (the ratio of IC_50_ values in HaCat to cancer cells (K562, PC3, SW620, Cack-1)) were calculated. We have selected the most promising benzofuran derivatives labeled **6** and **8** (Scheme 1) with the lowest IC_50_ (half-maximal inhibitory concentration) and the highest TI (therapeutical index) and tested their effect on ROS generation, IL 6 secretion and apoptosis induction. 

#### 3.3.2. Trypan Blue Exclusion Assay 

To evaluate the antiproliferative effects of the novel benzofurans derivatives the trypan blue exclusion assay was performed. Cells (HaCat, and K562) were seeded into 12-well plates (2 × 10^4^ cells/well). After 24 h, cells were exposed to the tested compounds at concentrations of their respective IC_50_ and incubated for 72h. Then, cells were harvested and centrifugated (500× *g*, 5min). After centrifugation supernatant was removed and cell sediment was dissolved in 0.2 mL of PBS. Next, 10 µL of cells solution was mixed with 10 µL of trypan blue dye. Cells were counted by automatic cell counter (Countessa). Data obtained were expressed as the mean ± SD and the mean percentage of viable cells was calculated from three independent experiments performed in triplicate. 

#### 3.3.3. Annexin V-FITC Binding Apoptosis Determination Assay

In order to evaluate apoptosis through the presence of phosphatidylserine on the extracellular side of the cellular membrane, FITC Annexin V Apoptosis Detection Kit I (BD Pharmingen) was used as a method of analysis. K562 and HaCat cells (3 × 10^4^/well) were seeded into 6-well plates and left to set for 24 h. Next, cells were treated with IC_50_ concentration of the most promising test compounds (compound **6** and **8**) and incubated for 72 h. The effects of compounds towards cell lines were observed by dual staining (Annexin V: FITC and PI. Annexin V: FITC) and for cellular DNA using PI according to the manufacturer’s instruction. Fluorescence sample containing 1 × 10^3^ cells were analyzed using flow cytometry (Becton Dickinson). Analyzed cell samples were divided into the following four populations: Q1, necrotic cells (Annexin V-FITC negative and PI positive); Q2, cells in the late stage of apoptosis (Annexin V-FITC and PI positive); Q3, control (viable) cells, not undergoing apoptosis or necrosis (Annexin V-FITC and PI negative); and Q4, early apoptotic cells (Annexin V-FITC positive and PI negative).

#### 3.3.4. Caspases 3 and 7 Activity Assay 

The induction of cell apoptosis was also analyzed by luminescent assay with the Caspases-Glo3/7 assay system (Promega). K562 and HaCaT cells (1 × 10^4^/well) were seeded onto a 96-well plate. After 24 h, cells were exposed to compounds **6** and **8** and their respective IC_50_ concentrations. Cells were incubated for 4, 6, and 12 h. After incubation, cells were treated with caspase 3/7 reagent (according to manufacturer’s instruction) and incubated for a further 1.5 h at room temperature. Fluorescents of the wells (performed three times for each well) were measured at the excitation wavelength of 485nm and emission wavelength of 520nm using a Microplate Spectrofluorometer BioTek Synergy™ (BioTek Instruments, Santa Clara, CA, USA).

#### 3.3.5. ROS Detection: DHR 123 and DCFH-DA 

ROS generation was performed using the spectrofluorometric method using dihydrorhodamine 123 (DHR 123) and 2′,7′-dichlorodihydrofluorescein diacetate (DCFH-DA). The following analysis is based on ROS-dependent oxidation of the compounds to fluorescent rhodamine 123 and dichlorofluorescein (DCF), respectively. K562 cells were seeded to a 96-well plate (1 × 10^4^ cells) and the selected benzofuran compounds (**6** and **8**) were added according to their respective IC_50_. The incubation times were 6 and 12 h, respectively. Then, the medium was removed, and cells were rinsed with PBS and incubated with DHR 123 (5M) and DCFH-DA (5M) for 60 min at 37 °C in the dark. In order to obtain a positive and negative control, a sample with H_2_O_2_ (0.25 mM) and a sample without any reagent were prepared, respectively. The maximum excitation and emission spectra for rhodamine 123 were 500 nm and 536 nm, respectively, and for DCF were 492 nm and 527 nm, respectively. The generation of H_2_O_2_ was measured by a Microplate Spectrofluorometer BioTek Synergy™ (BioTek Instruments, USA) and expressed as DCF fluorescence intensity (FI) and rhodamine fluorescence intensity (FI). The resulting data were expressed as mean ± SD from three independent experiments performed in triplicate. 

#### 3.3.6. Interleukin-6

The level of IL-6 was measured by ELISA (Diaclon SAS, Besancon, France). K562 cells were treated with IC_50_ concentrations of the tested compounds. After 72 h, medium was collected and the IL-6 level was measured using an enzyme-linked immunosorbent assay, in accordance with the manufacturer’s protocol. The resulting data were expressed as mean ± SD from three independent experiments performed in triplicate. 

### 3.4. Antimicrobial Activity 

The antimicrobial studies for all of the obtained compounds were conducted using reference strains of bacteria derived from international microbe collections—the American Type Culture Collection (ATCC) and the National Collection of Type Culture (NCTC). Among the reference strains there were two Gram-negative rods (*P. aeruginosa* ATCC 15442 and *E. coli* ATCC 25922) and six Gram-positive isolates (*Staphylococcus aureus:* NCTC 4163, ATCC 25923, ATCC 6538, ATCC 29213, *S. epidermidis* ATCC 12228, ATCC 35984). These strains are widely used to determine the activity of antimicrobials and check the drug susceptibility of bacteria, and their use enables comparison of assay results obtained in independent laboratories all over the world. 

The antimicrobial activity of compounds was expressed by minimum inhibitory concentration values (MICs), according to CLCI reference procedures with some modifications. MIC was tested by the two-fold serial microdilution method (in 96-well microtiter plates) on MH II liquid medium. The final inoculum of all of the studied bacteria was 10^6^ CFU/mL (colony forming unit per mL). The stock solution of tested compounds was prepared in DMSO and was diluted in sterile medium (to a maximum of 1% of solvent content). The concentrations of compounds were from 16 to 512 µg/mL. The MIC value was the lowest concentration of the compound at which bacterial growth was no longer observed after 18 h of incubation at 35 °C. Clinical microorganisms used in this study were obtained from the collection of the Department of Pharmaceutical Microbiology, Medical University of Warsaw, Poland [70,71]. 

### 3.5. Statistical Analysis

The statistical calculation was performed using Statistica 13.1 (StatSoft, Inc., Tulsa, OK, USA) program. The quantitative comparisons were made using Dunnett test. The IC_50_ values were estimated by GraphPad Prism 8.0.1. Results of all presented experiments were expressed as the mean ± SD and considered statistically significant at *p* < 0.05.

## 4. Conclusions

In summary, we have identified two novel halogen derivatives of benzofurans (referred to as **6** and **8**) that exhibit selective cytotoxic effects against leukemia cancer cells (K562). Significantly, both compounds displayed lower toxicity toward normal cells (HaCaT), resulting in a favorable therapeutic index (TI). Our research has demonstrated that both of these compounds induce apoptosis in the examined cancer cells by increasing the activity of executioner caspases 3/7. Furthermore, our findings suggest that the antitumor potential of these selected derivatives may be linked to an enhanced production of reactive oxygen species (ROS) and the inhibition of interleukin-6 (IL-6) release.

In conclusion, it appears that bromo derivatives of benzofurans hold significant promise as potential anticancer agents targeting leukemia cancer cells. These findings are consistent with the results of our previously documented studies.

## Figures and Tables

**Table 1:** Cytotoxic activity (IC_50_, μM) of the studied compounds estimated by the MTT assay.

Compound	K562 ^a^	PC3 ^b^	SW620 ^c^	Cacki-1 ^d^	HaCaT ^e^	TI ^f^
**1**	57.00 ± 3.50	80 ± 20.0	>100	>100	>100	>100
**2**	36.32 ± 4.5	>100	>100	>100	67.30 ± 29.68	1.853
**3**	58.00 ± 21.12	66.41 ± 28.72	52.33 ± 22.5	>100	>100	1.724
**4**	60.00 ± 9.0	>100	>100	>100	55.29 ± 33.44	0.925
**5**	10.30 ± 0.55	17.50 ± 3.5	15.60 ± 4.1	>100	15.00 ± 0.80	1.450
**6**	3.83 ± 0.60	7.02 ± 2.22	>100	>100	12.44 ± 1.27	3.248
**7**	50.00 ± 10.0	40.00 ± 15.0	47.15 ± 22.25	>100	5.69 ± 5.67	0.114
**8**	2.59 ± 0.88	7.86 ± 2.62	>100	>100	23.57 ± 10.7 0	9.100
**9**	14.40 ± 3.70	40.00 ± 20.0	>100	>100	22.80 ± 1.51	1.583
Bortezomib	0.04 ± 0.01	nd	nd	nd	nd	nd
Doxorubicin	0.21 ± 0.10	nd	nd	nd	nd	nd

Data are expressed as mean ± SD from three independent experiments. IC_50_ (µM)—the concentration of the compound that corresponds to a 50% growth inhibition of cell lines (as compared with the control) after cells are incubated with tested compounds. ^f^—Therapeutic index (TI^f^)—the ratio of IC50 values in HaCat to cancer cells (K562). **^a^**—Human chronic myelogenous leukemia (K562), **^b^**—human metastatic prostate cancer (PC3),**^c^**—human metastatic colon cancer, **^d^**—human clear cell renal cell carcinoma, **^e^**—human immortal keratinocytes cell line from adult human skin (HaCaT). nd: not determined.

**Table 2:** The effect of compounds **6** and **8** on the total cell number and viability (%) in K562, and HaCaT measured by trypan blue assay.

	Compound	Cell Number (×10^6^)	Percentage of Viability (%)
**HaCaT ^a^**	Control	2.70 ± 0.10	93.0 ± 0.27
	**6**	2.45 ± 1.63	80.5 ± 19.09
	**8**	2.00 ± 0.42	87.0 ± 8.70
**K562 ^b^**	Control	0.75 ± 0.09	91.5 ± 3.50
	**6**	0.62 ± 0.12	94.0 ± 1.00
	**8**	0.83 ± 0.12	96.0 ± 1.41

Data are expressed as mean ± SD; ^a^—human immortal keratinocytes cell line from adult human skin (HaCaT ), ^b^—human chronic myelogenous leukemia (K562).

**Table 3:** The effect of derivatives **6** and **8** on IL-6 concentration (mean ± SD), measured by ELISA kit. K562 cells were incubated with studied compounds for 72 h at their IC_50_ concentrations. *** *p* ≤ 0.001 as compared with the control cells.

	Il-6 Concentration in K562 Cells in 72 h
Control	11.616 ± 1.730997
**6**	5.791 ± 2.047781 ***
**8**	6.958 ± 3.622508 ***

**Table 4:** The activity of compound **7** against standard bacteria strains, expressed by minimal inhibitory concentrations (µg/mL).

Strain	Compound 7 (µg/mL)	Ciprofloxacine (µg/mL)
*S.aureus NCTC 4163*	>512	0.25
*S.aureus ATCC 25923*	16	0.5
*S.aureus ATCC 6538*	64	0.125
*S.aureus NCTC 29213*	32	0.5
*S. epiderminis ATCC 122228*	16	0.125
*S. epidermidis ATCC 35984*	16	0.125
*E. coli ATCC 25922*	>512	0.015
*P. aeruginosa ATCC 15442*	>512	0.06

## Data Availability

Data will be made available on request.

## Supplementary Materials

Supplementary materials can be found at https://www.mdpi.com/article/10.3390/ijms25041999/s1.
