# Supplementary material for: New Derivatives of 1-(3-Methyl-1-Benzofuran-2-yl)Ethan-1-one: Synthesis and Preliminary Studies of Biological Activity
Source: Int J Mol Sci. 2024 Feb 7;25(4):1999. doi: 10.3390/ijms25041999 (PMC10888192; doi:10.3390/ijms25041999)

Supplementary Material

New Derivatives of 1-(3-Methyl-1-Benzofuran-2-yl)Ethan-1-one: Synthesis and Preliminary Studies of Biological Activity

Mariola Napiórkowska [1,*], Pratheeba Kumaravel [1], Mithulya Amboo Mahentheran [1], Ewelina Kiernozek-Kalińska [2] and Emilia Grosicka-Maciąg [3]

1 Chair and Department of Biochemistry, Medical University of Warsaw, 1 Banacha Str., 02-097 Warsaw, Poland; s079330@student.wum.edu.pl (P.K.); mithulya.amboo.5@gmail.com (M.A.M.)

2 Department of Immunology, Faculty of Biology, University of Warsaw, 1 Miecznikowa Str., 02-096 Warsaw, Poland; e.kiernozek@uw.edu.pl

3 Department of Biochemistry and Laboratory Diagnostic, Collegium Medicum Cardinal Stefan Wyszyński University, Kazimierza Wóycickiego 1 Str., 01-938 Warsaw, Poland; e.grosicka-maciag@uksw.edu.pl

* Correspondence: mariola.napiorkowska@wum.edu.pl; Tel.: +48-(22)-572-06-93, Fax: +48-(22)-572-06-79

Spectrum 1. ^{1}HNMR of compound **1** (300 MHz, $CDCl_3$).

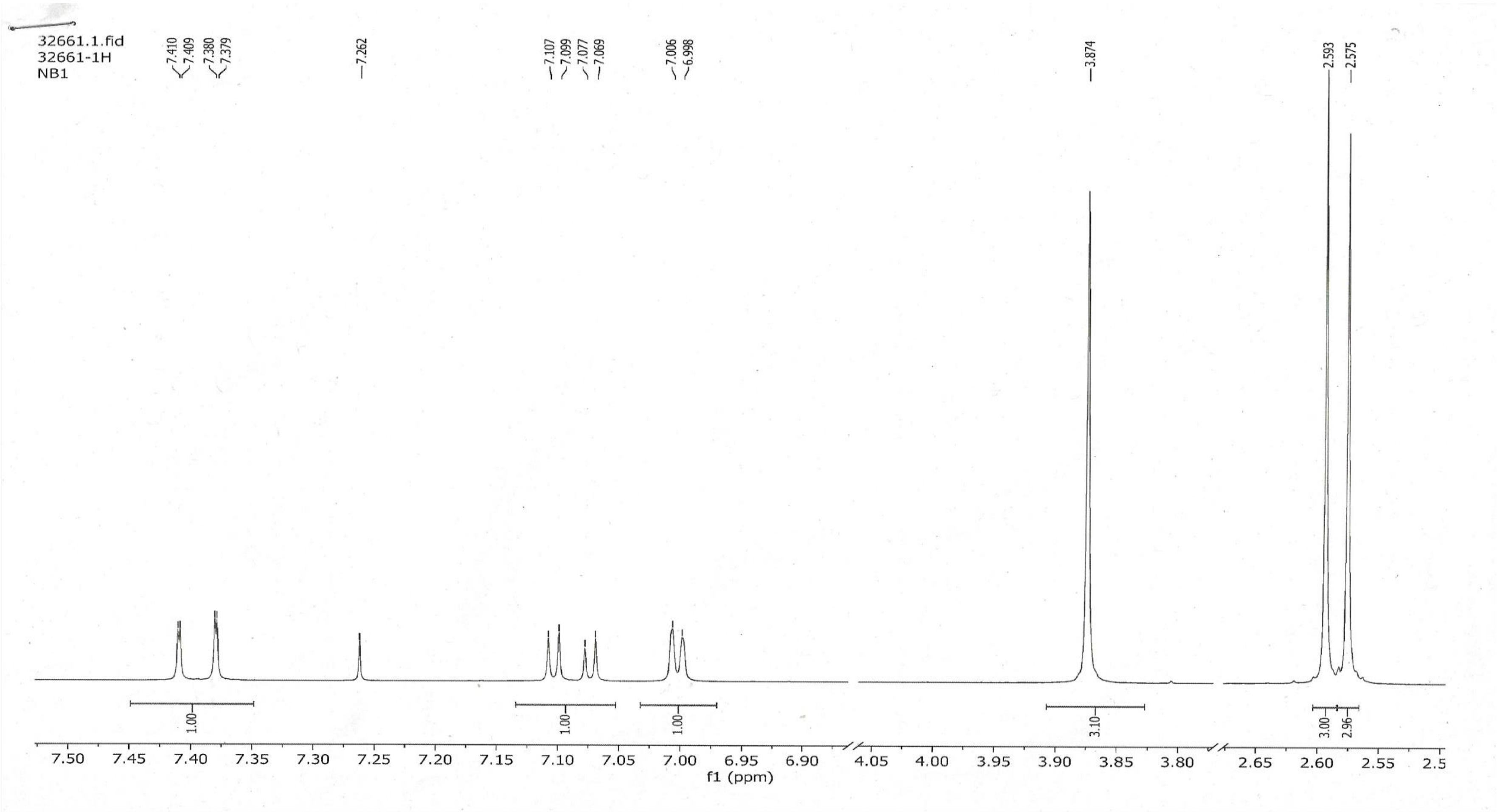

Spectrum 2. ^{1}HNMR of compound **2** (300 MHz, $CDCl_3$).

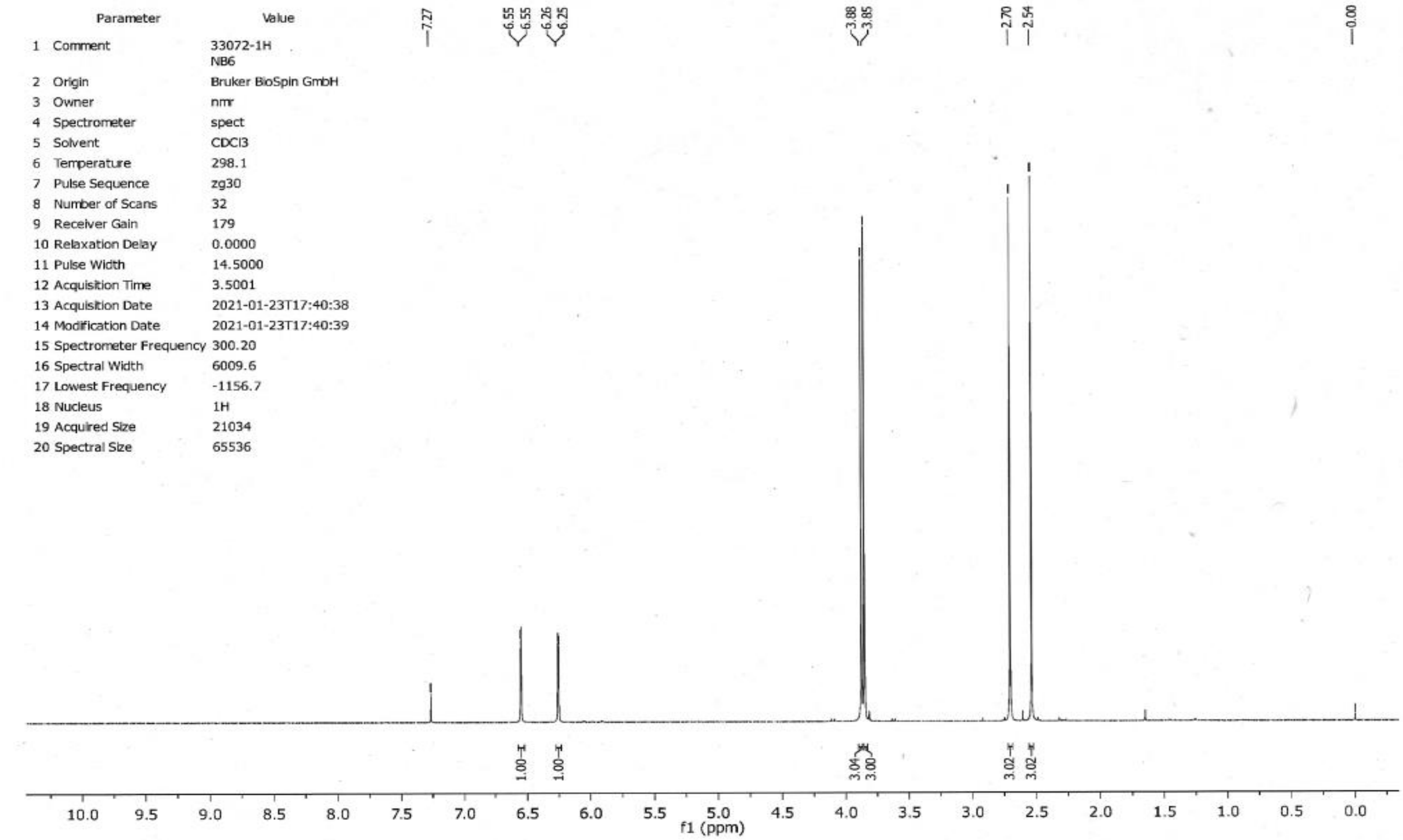

Spectrum 3. ^{1}HNMR of compound 3 (300 MHz, $CDCl_3$).

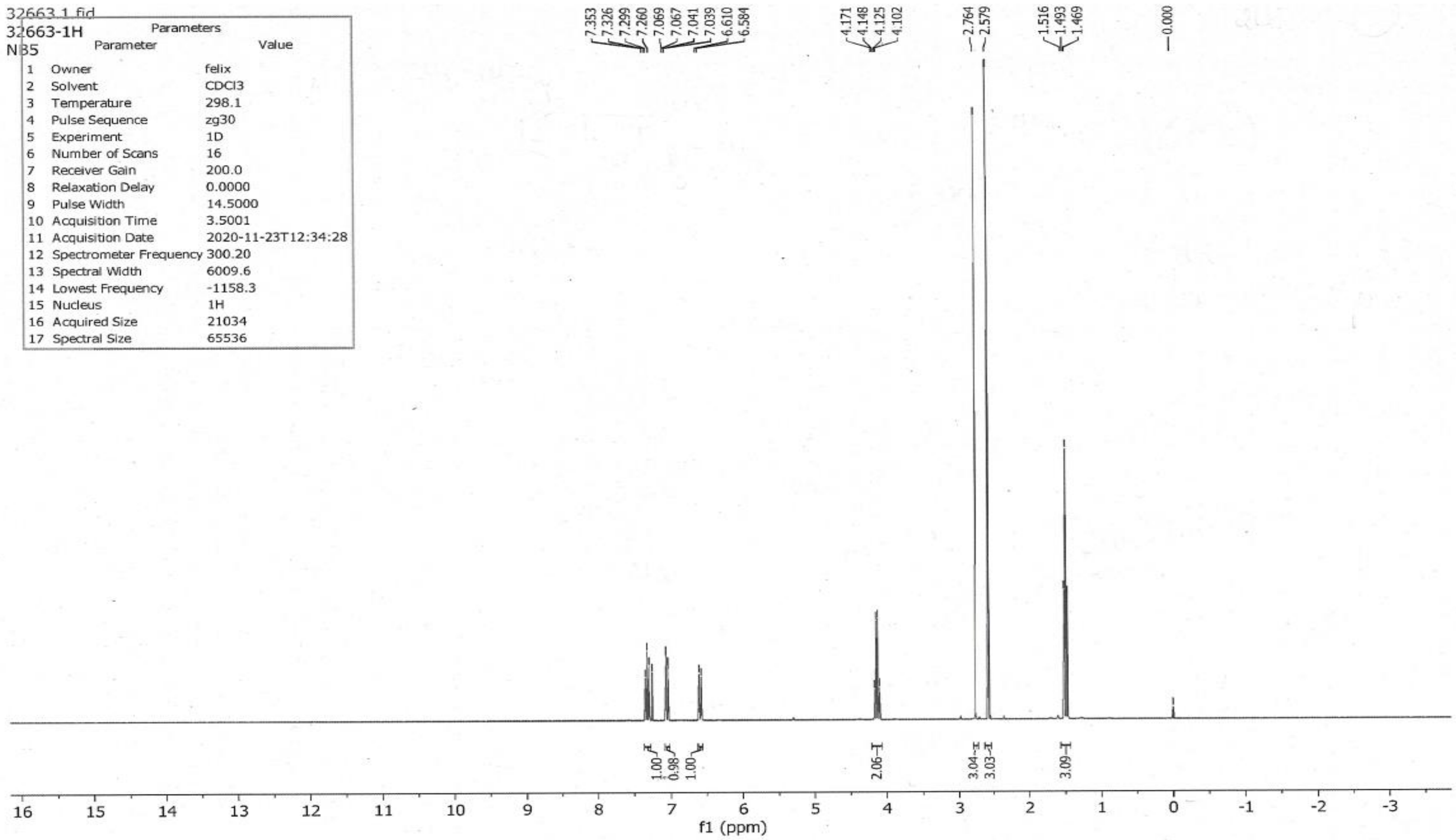

Spectrum 4. ^{1}HNMR of compound **4** (300 MHz, $CDCl_3$).

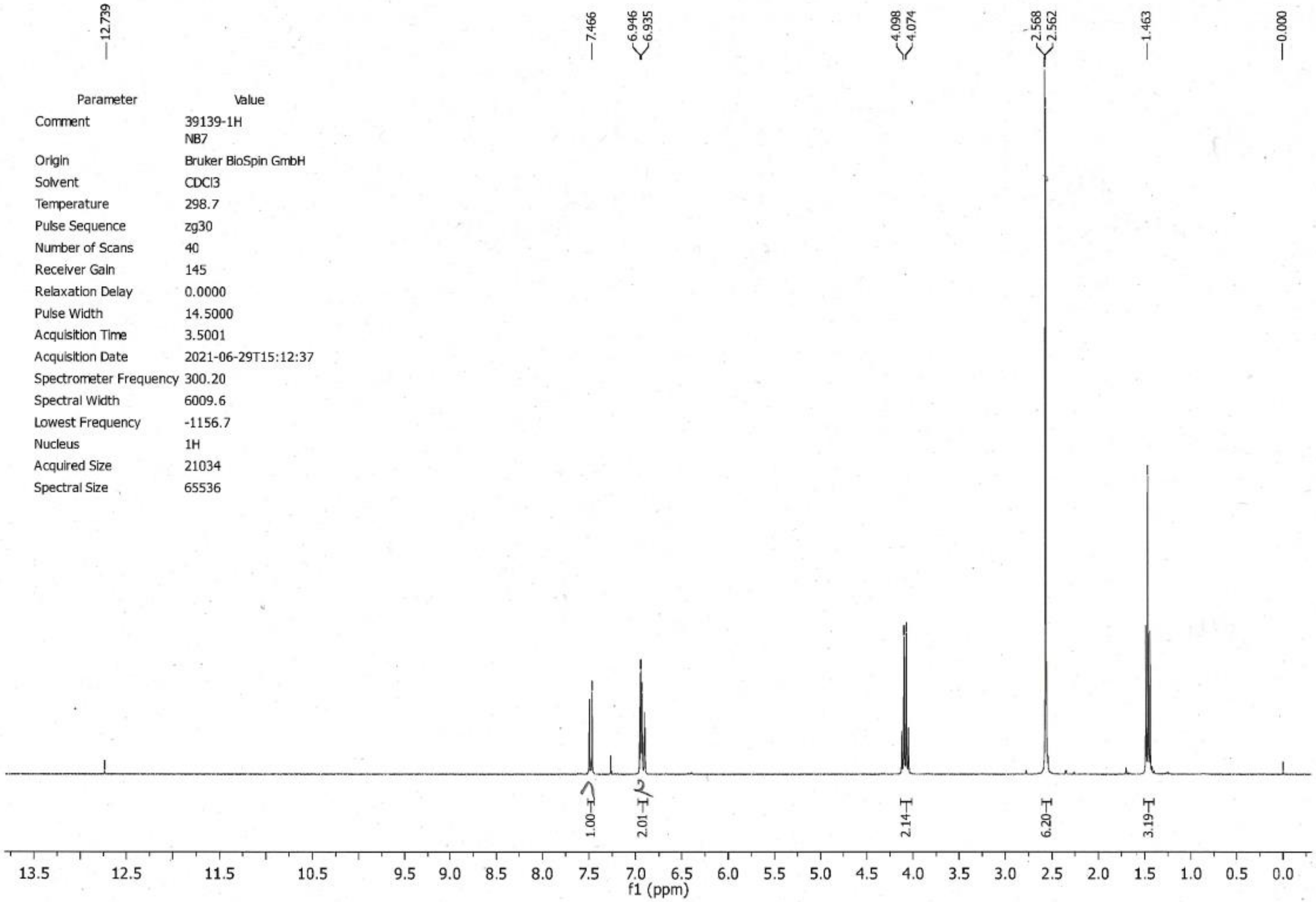

Spectrum 5. ^{1}HNMR of compound 5 (300 MHz, $CDCl_3$).

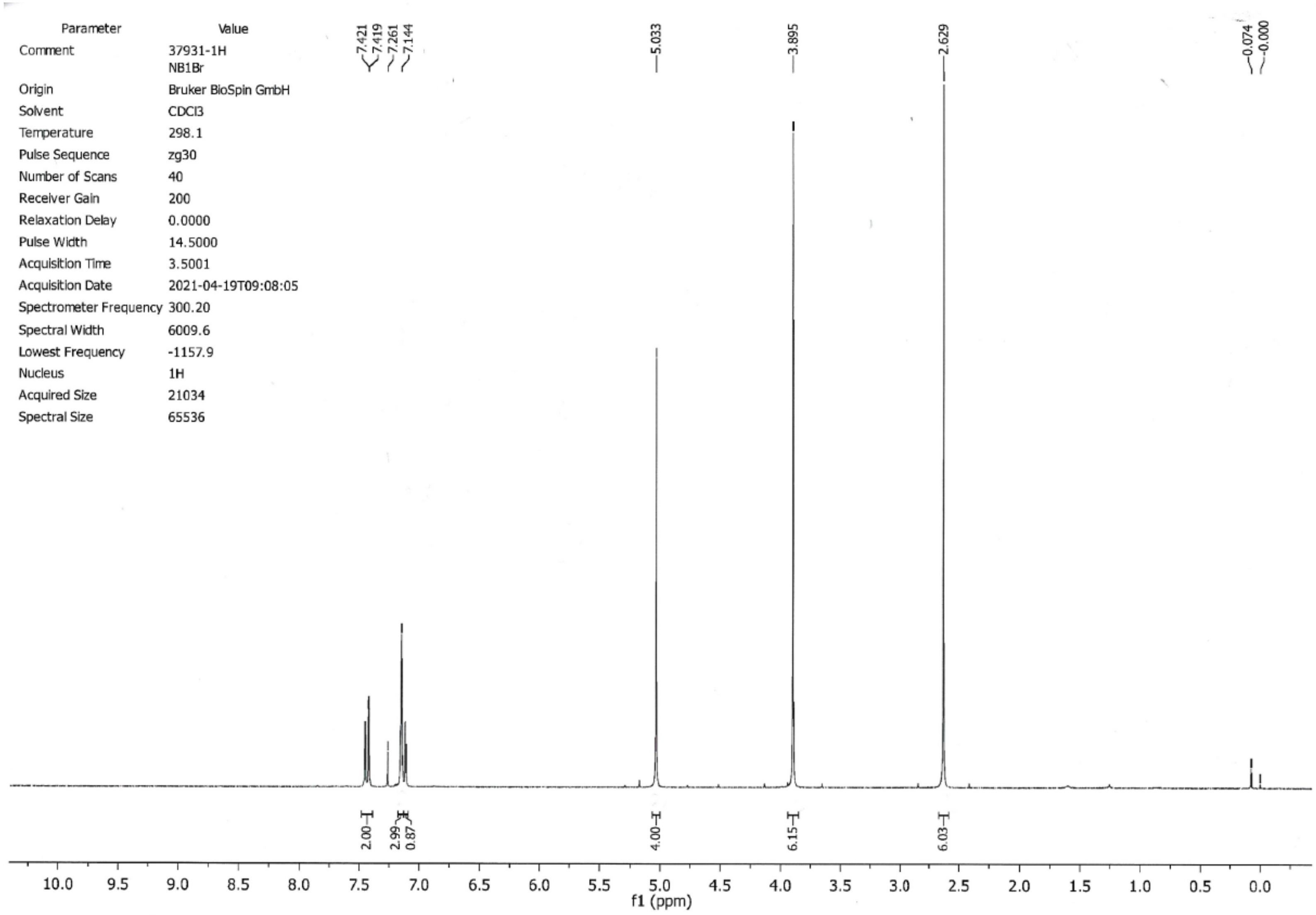

Spectrum 6. ^{1}HNMR of compound **6** (300 MHz, $CDCl_3$).

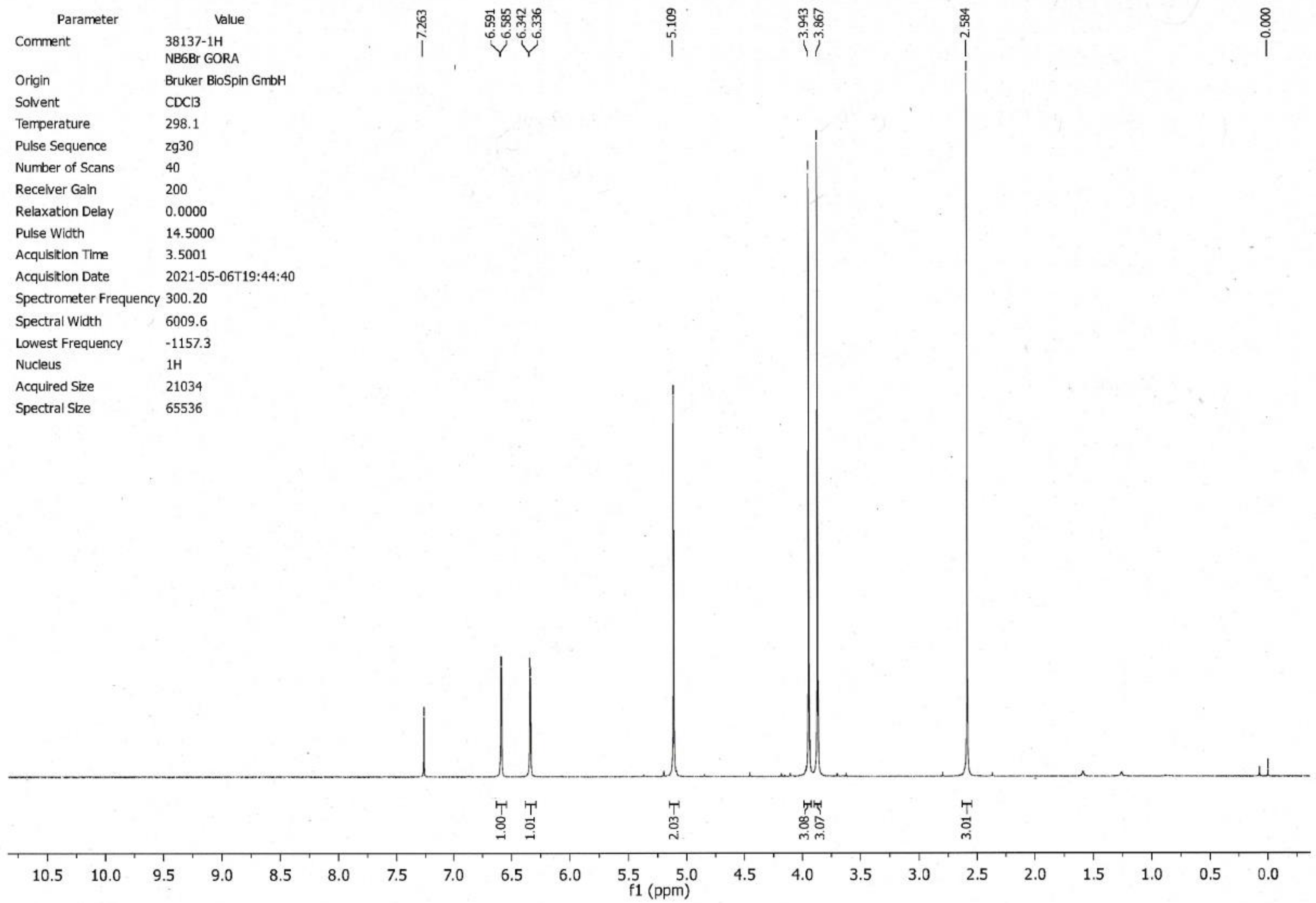

Spectrum 7. ^{1}HNMR of compound **7** (300 MHz, $CDCl_3$).

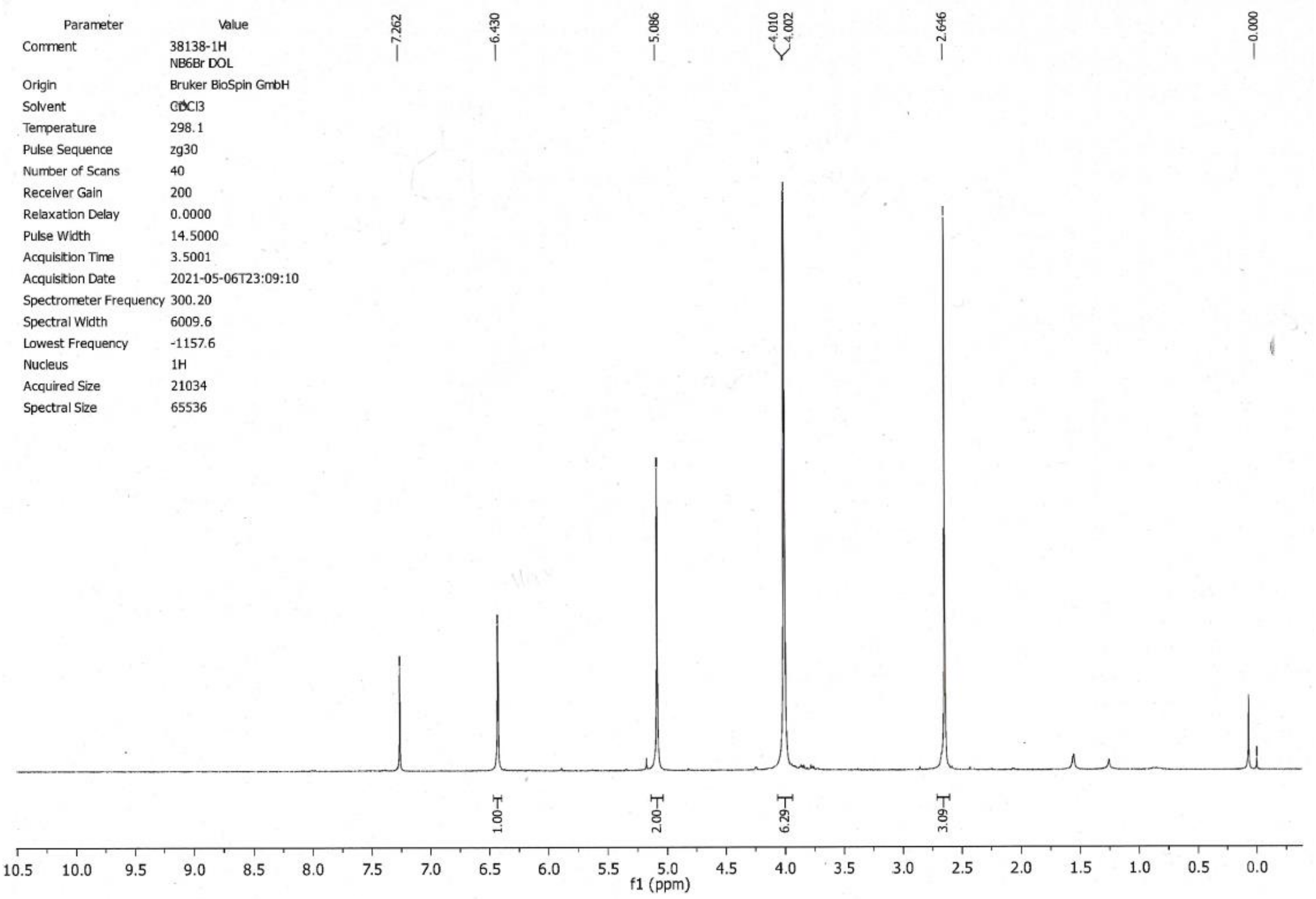

Spectrum 8. ^{1}HNMR of compound **8** (300 MHz, $CDCl_3$).

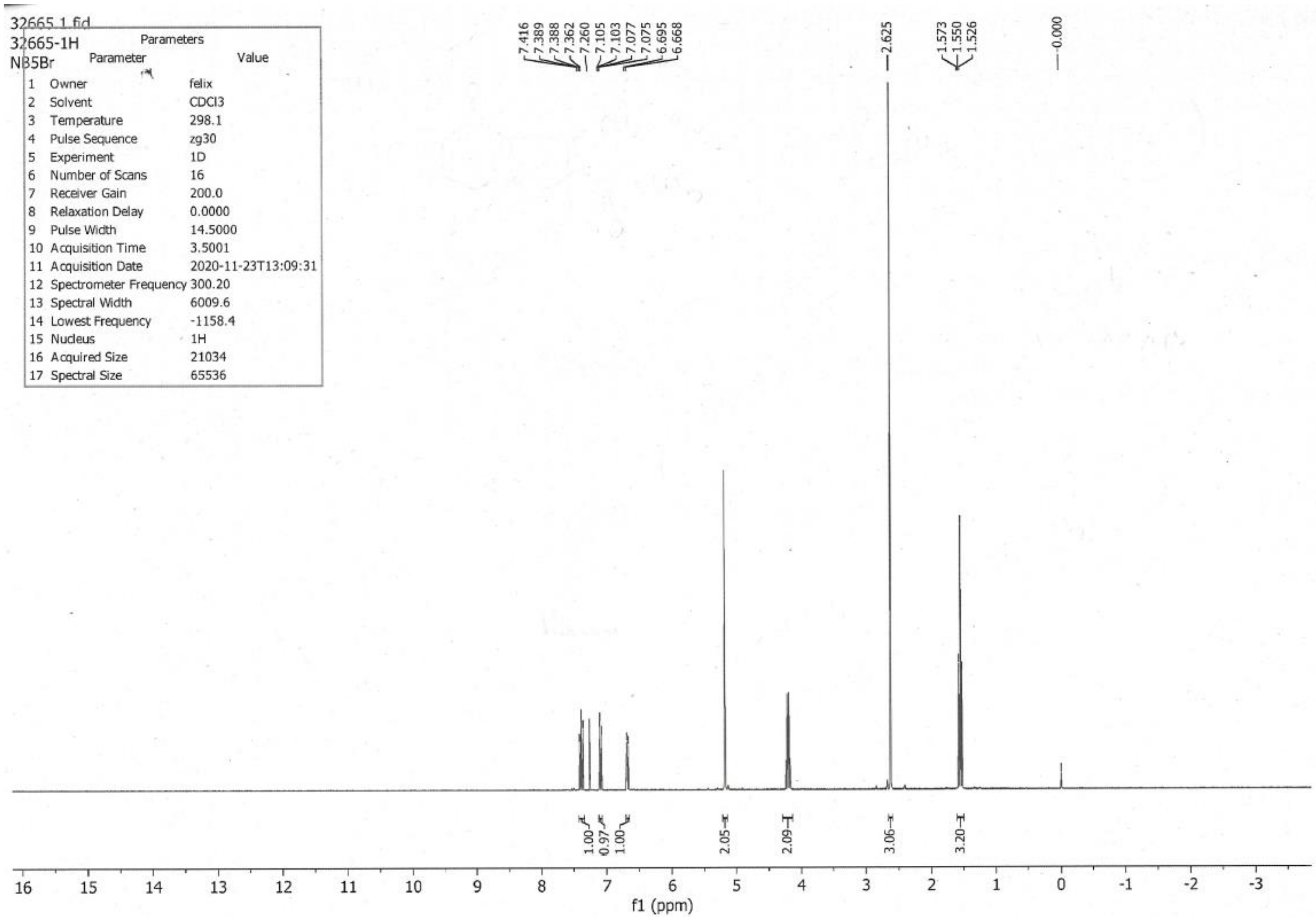

Spectrum 9. ^{1}HNMR of compound **9** (300 MHz, $CDCl_3$).

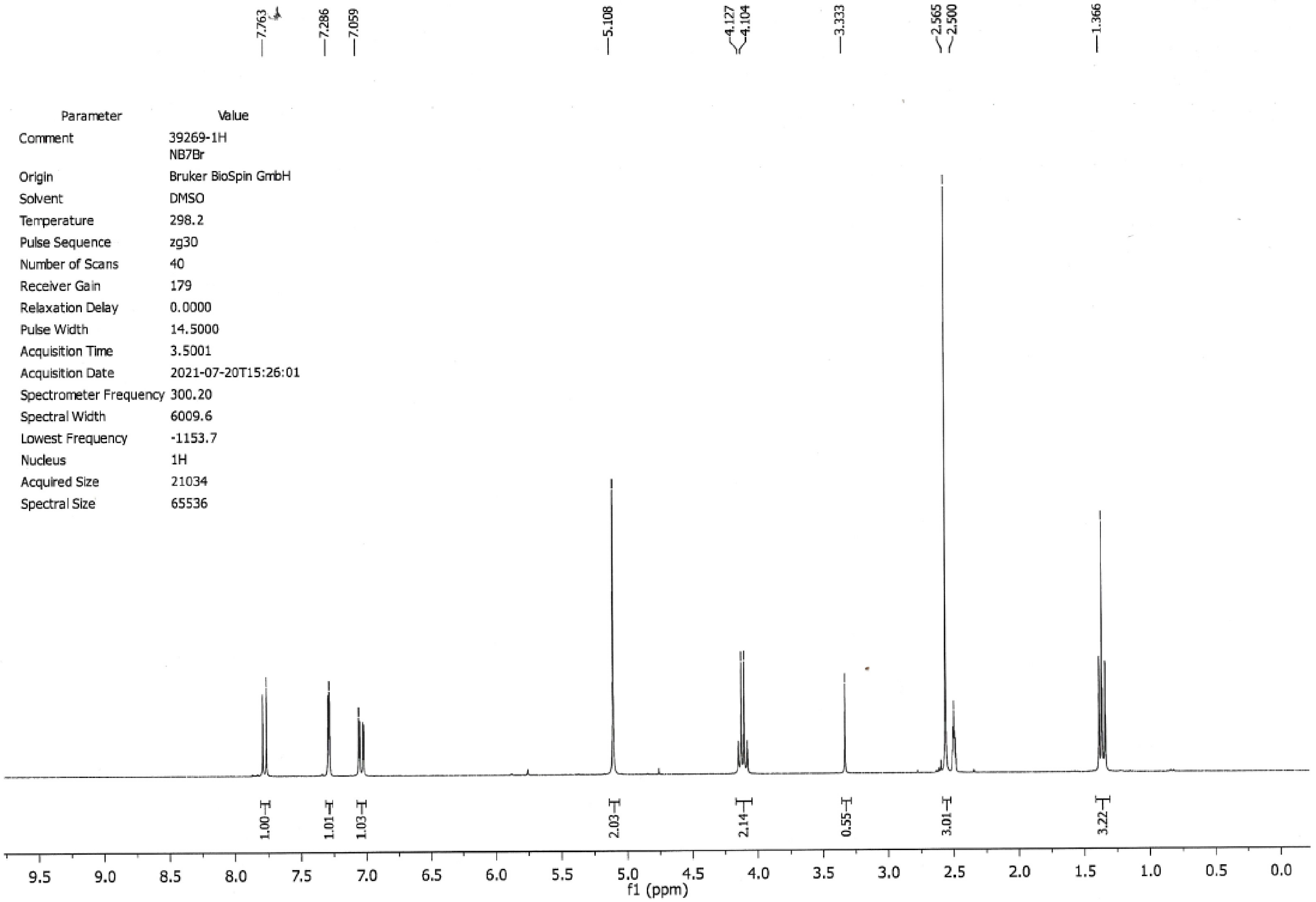

Spectrum 10. ^{13}C NMR of compound **1** (75.5 MHz, $CDCl_3$).

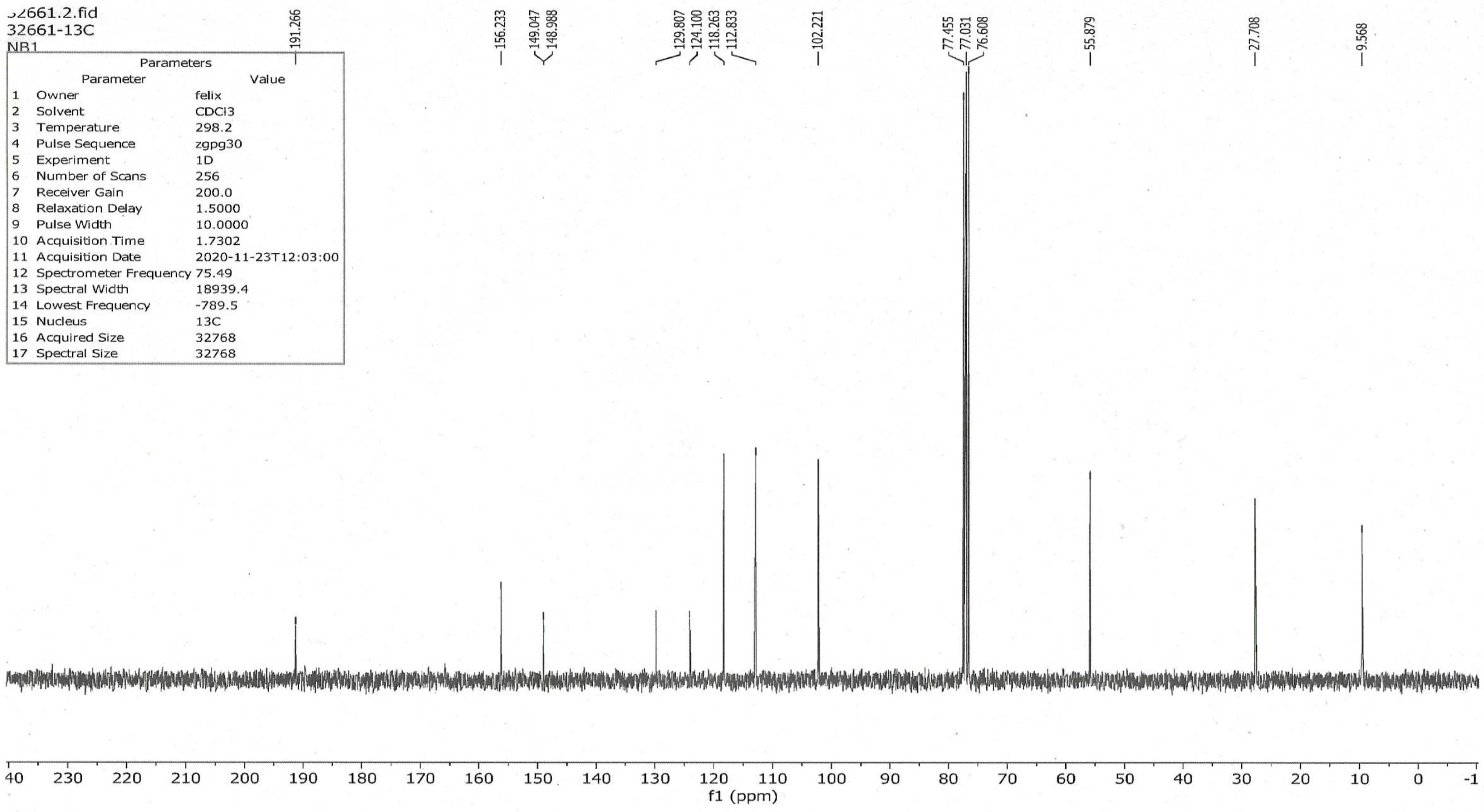

Spectrum 11. ^{13}C NMR of compound **2** (75.5 MHz, $CDCl_3$)

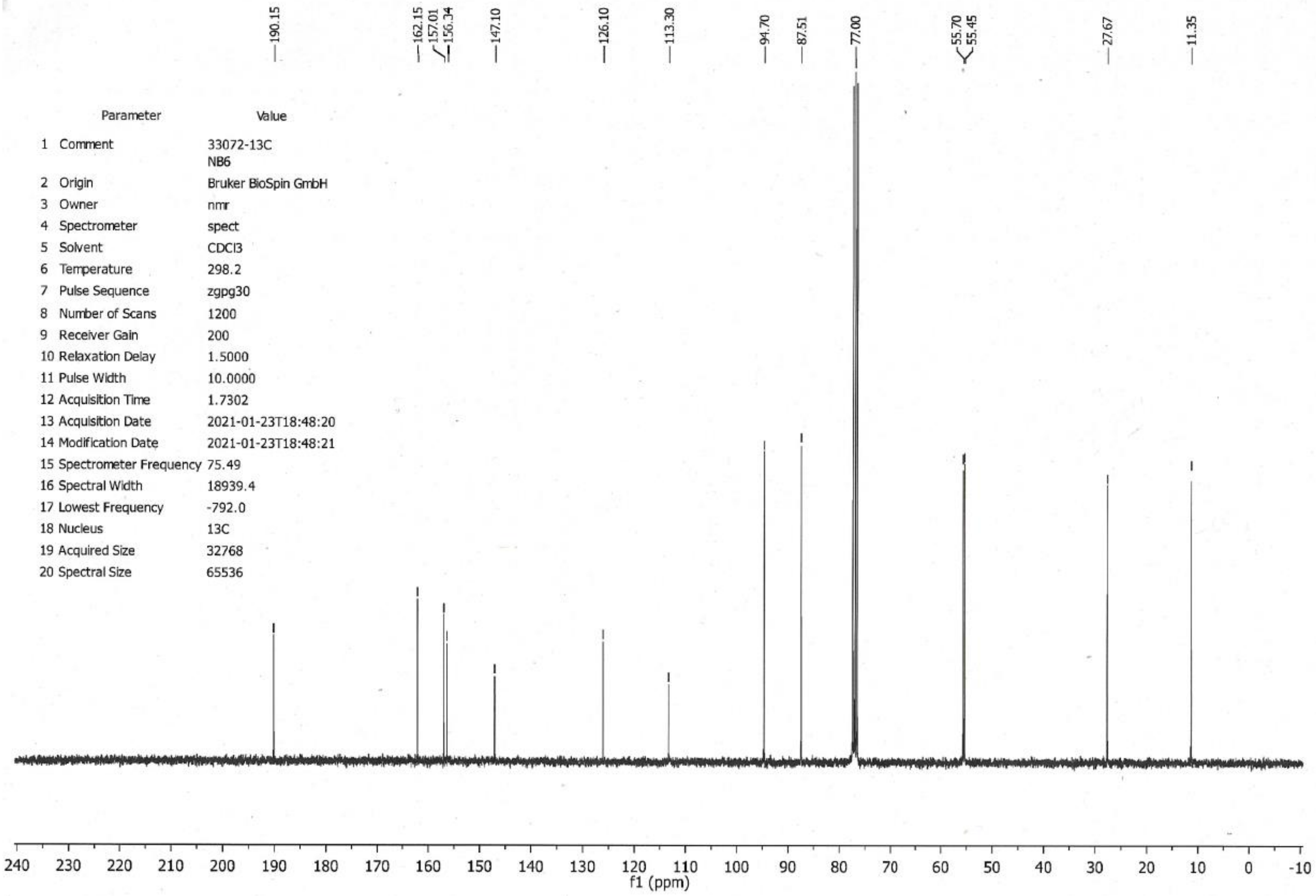

Spectrum 12. ^{13}C NMR of compound **3** (75.5 MHz, $CDCl_3$)

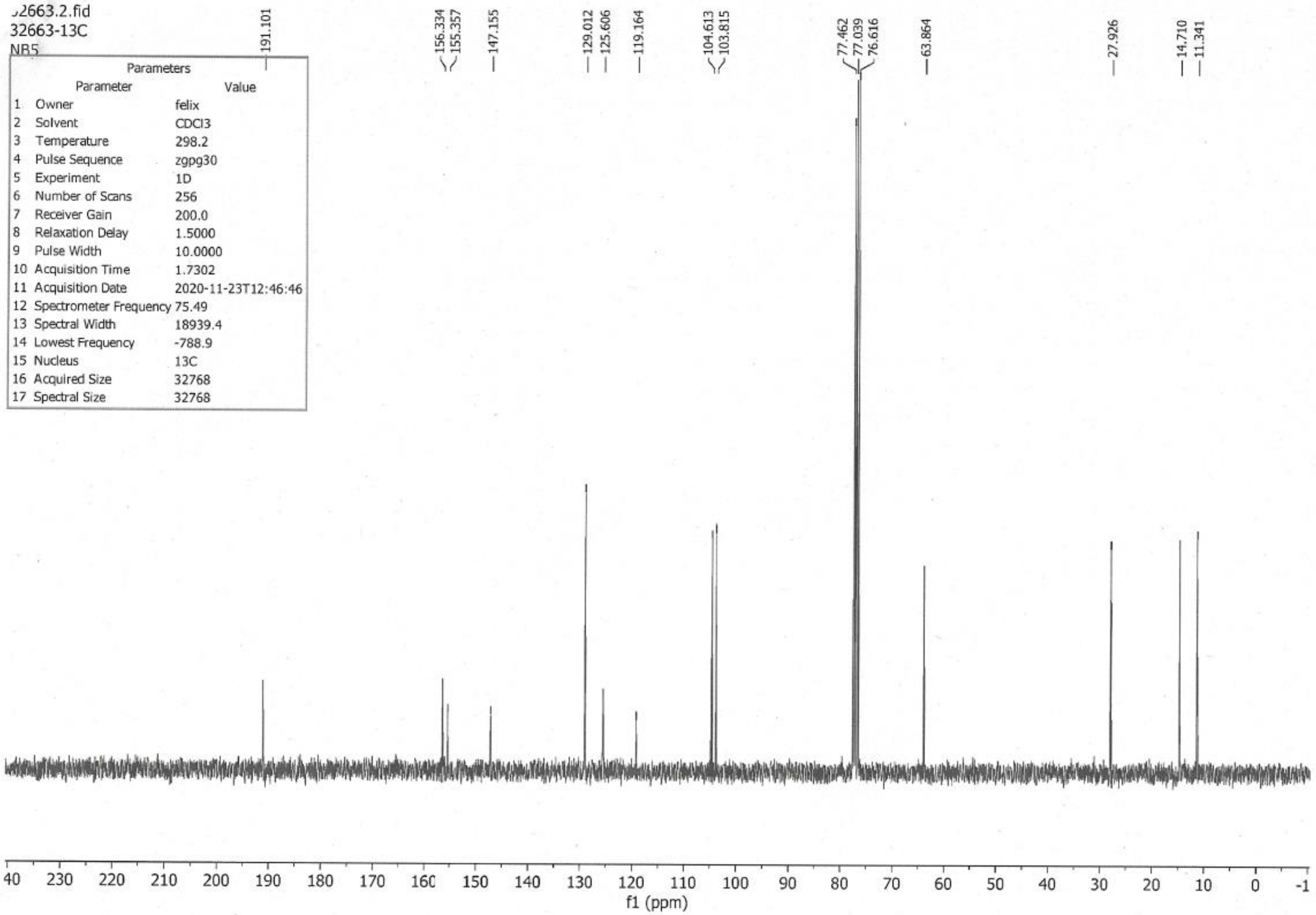

Spectrum 13. ^{13}C NMR of compound **4** (75.5 MHz, $CDCl_3$)

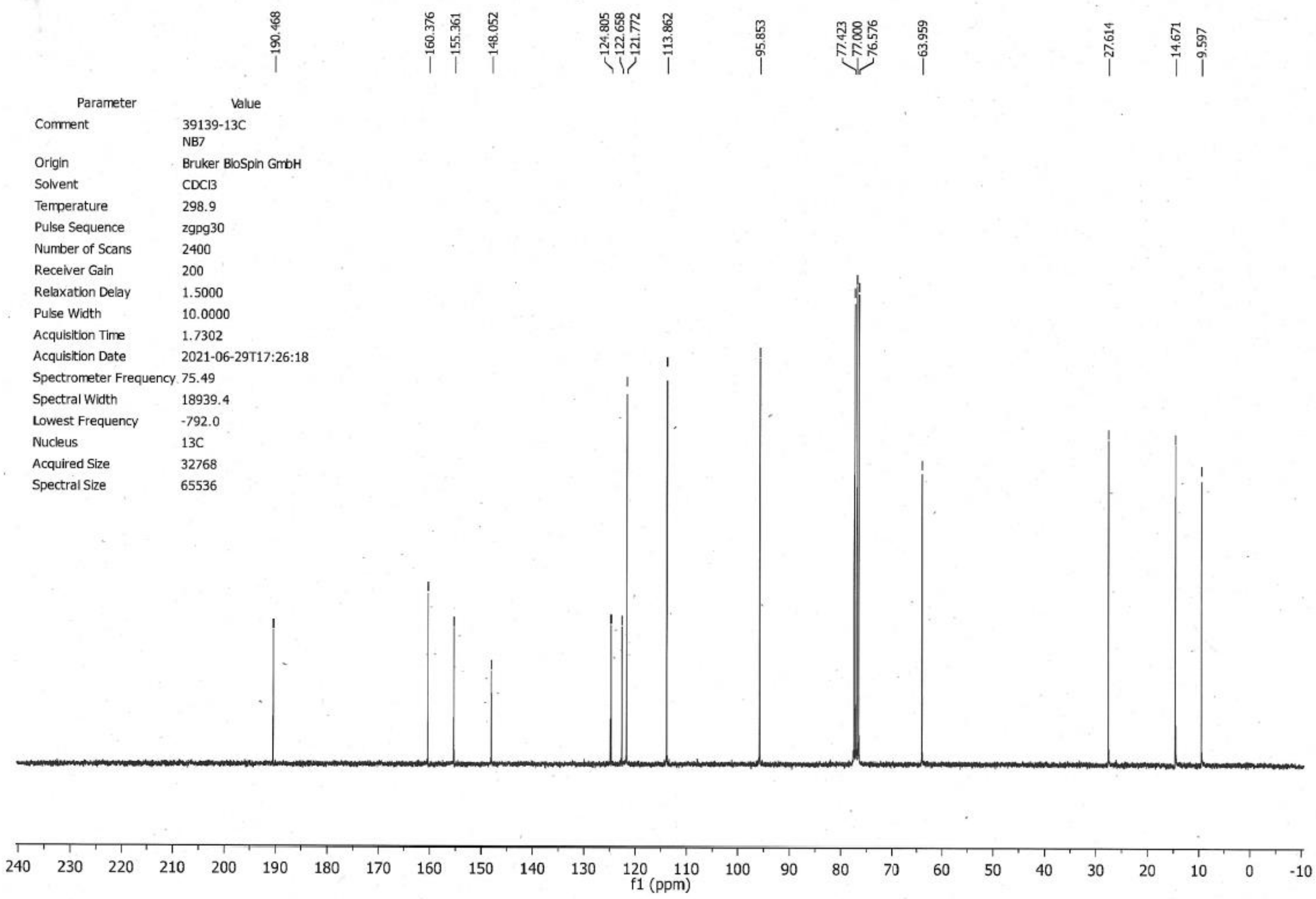

Spectrum 14. ^{13}C NMR of compound **5** (75.5 MHz, $CDCl_3$)

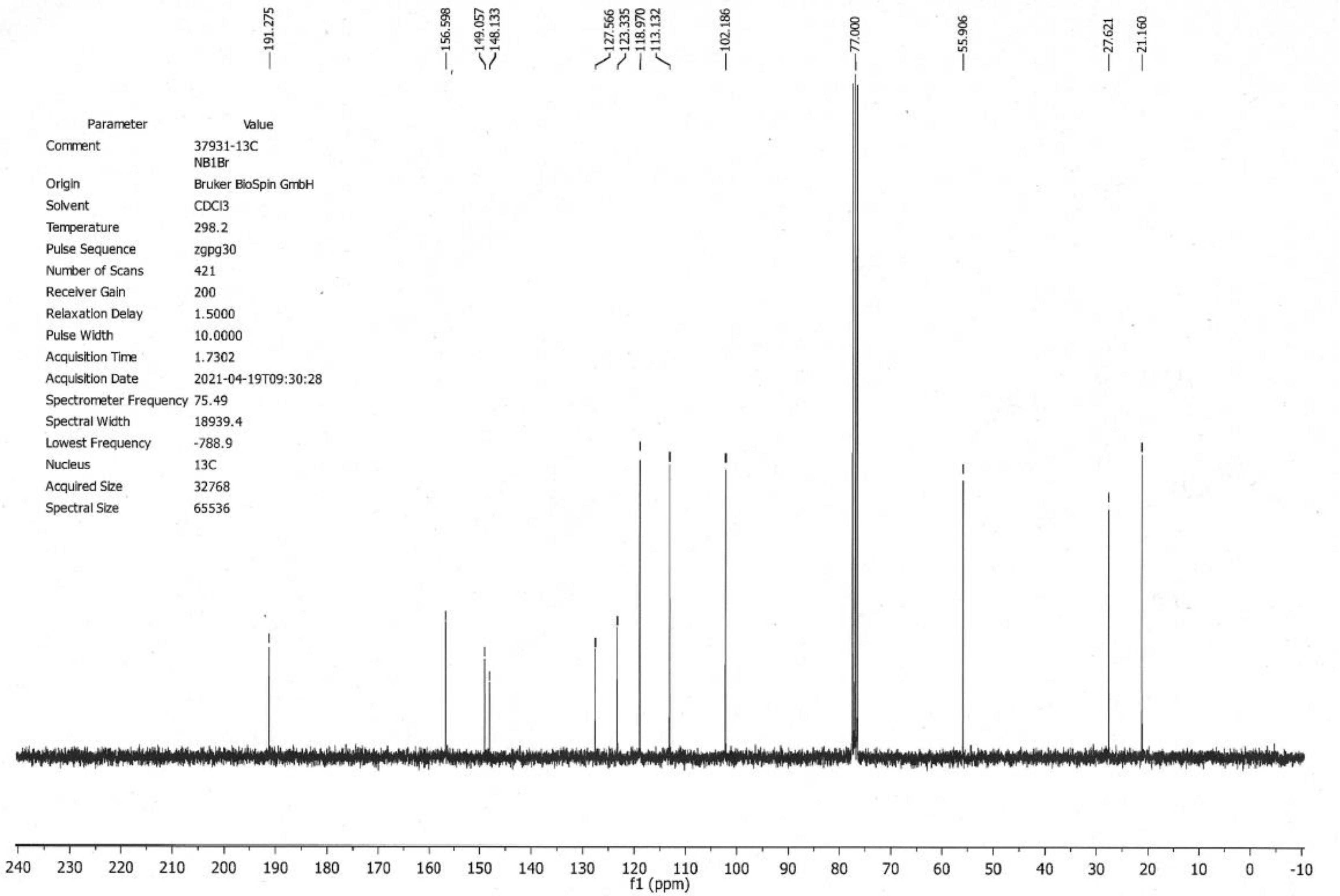

Spectrum 15. ^{13}C NMR of compound **6** (75.5 MHz, $CDCl_3$)

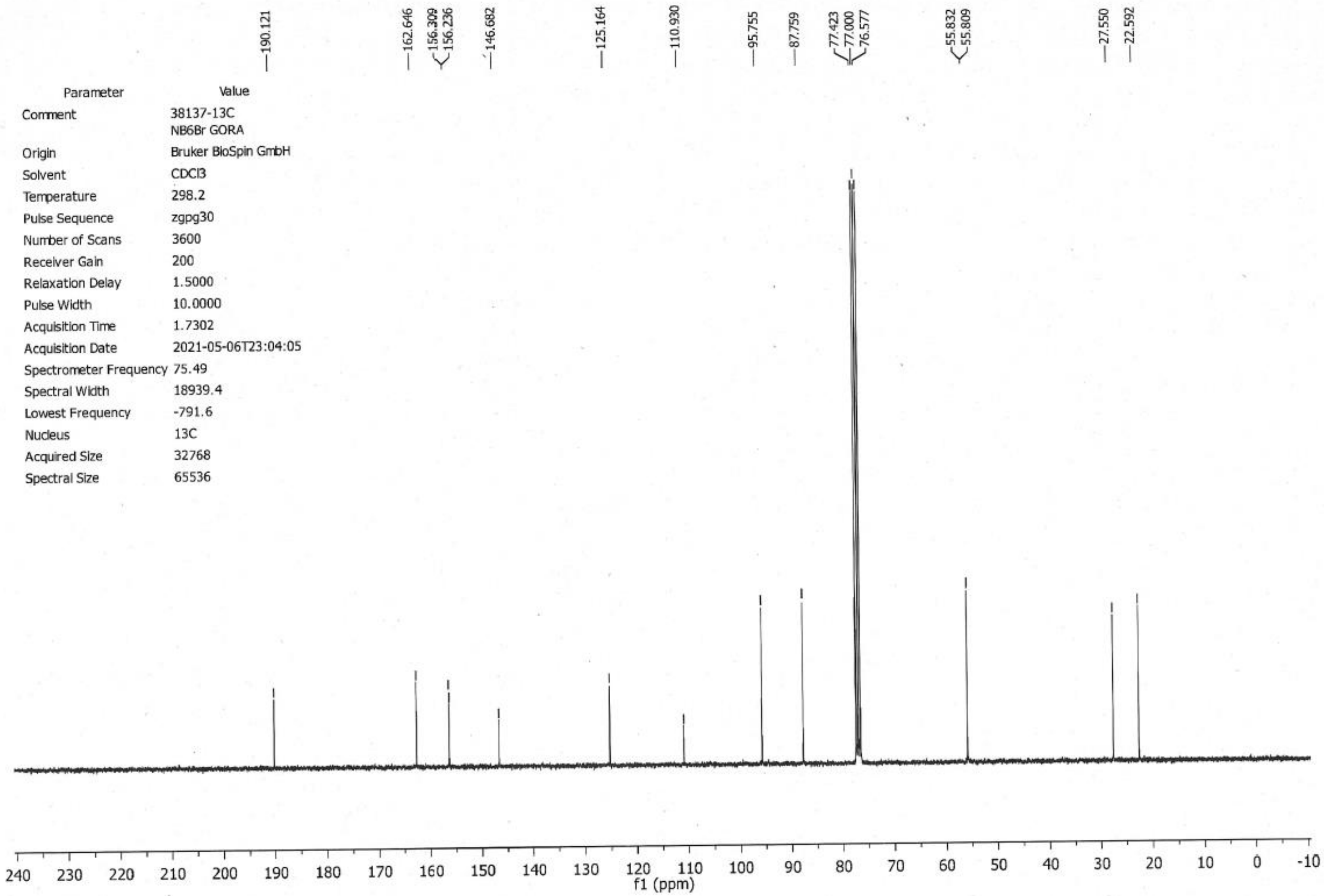

Spectrum 16. ^{13}C NMR of compound **7** (75.5 MHz, $CDCl_3$)

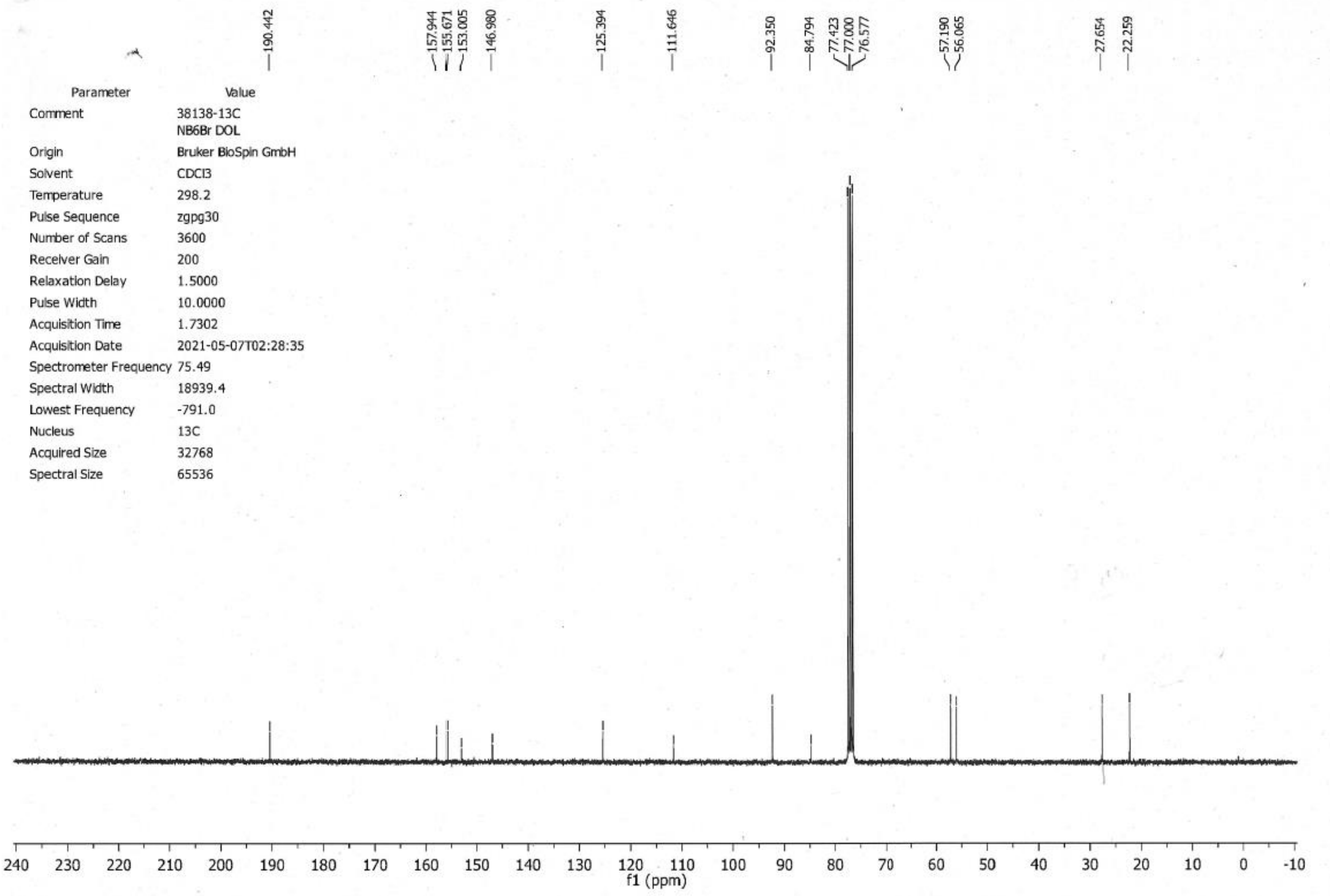

Spectrum 17. ^{13}C NMR of compound **8** (75.5 MHz, $CDCl_3$)

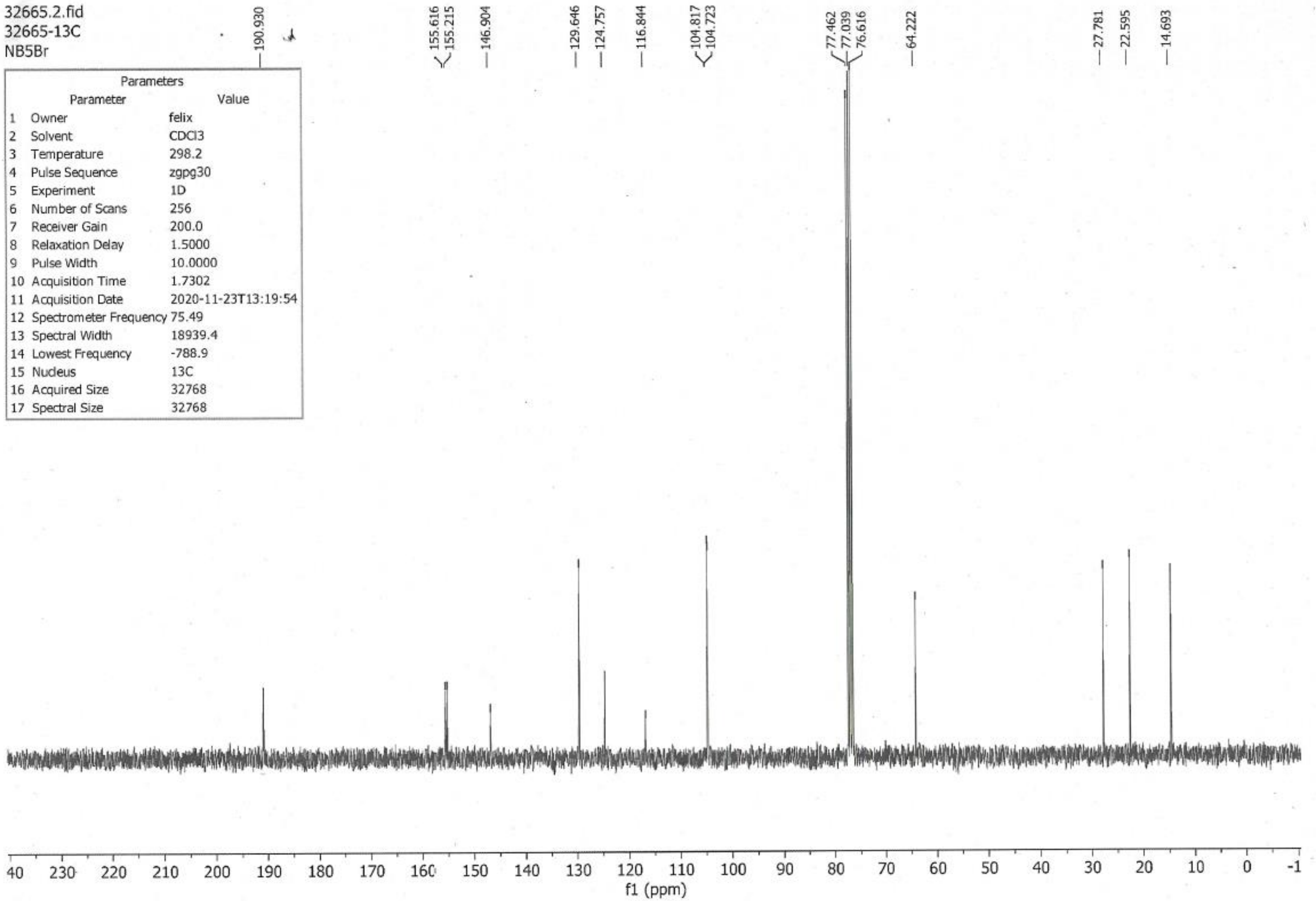

Spectrum 18. ^{13}C NMR of compound **9** (75.5 MHz, $CDCl_3$)

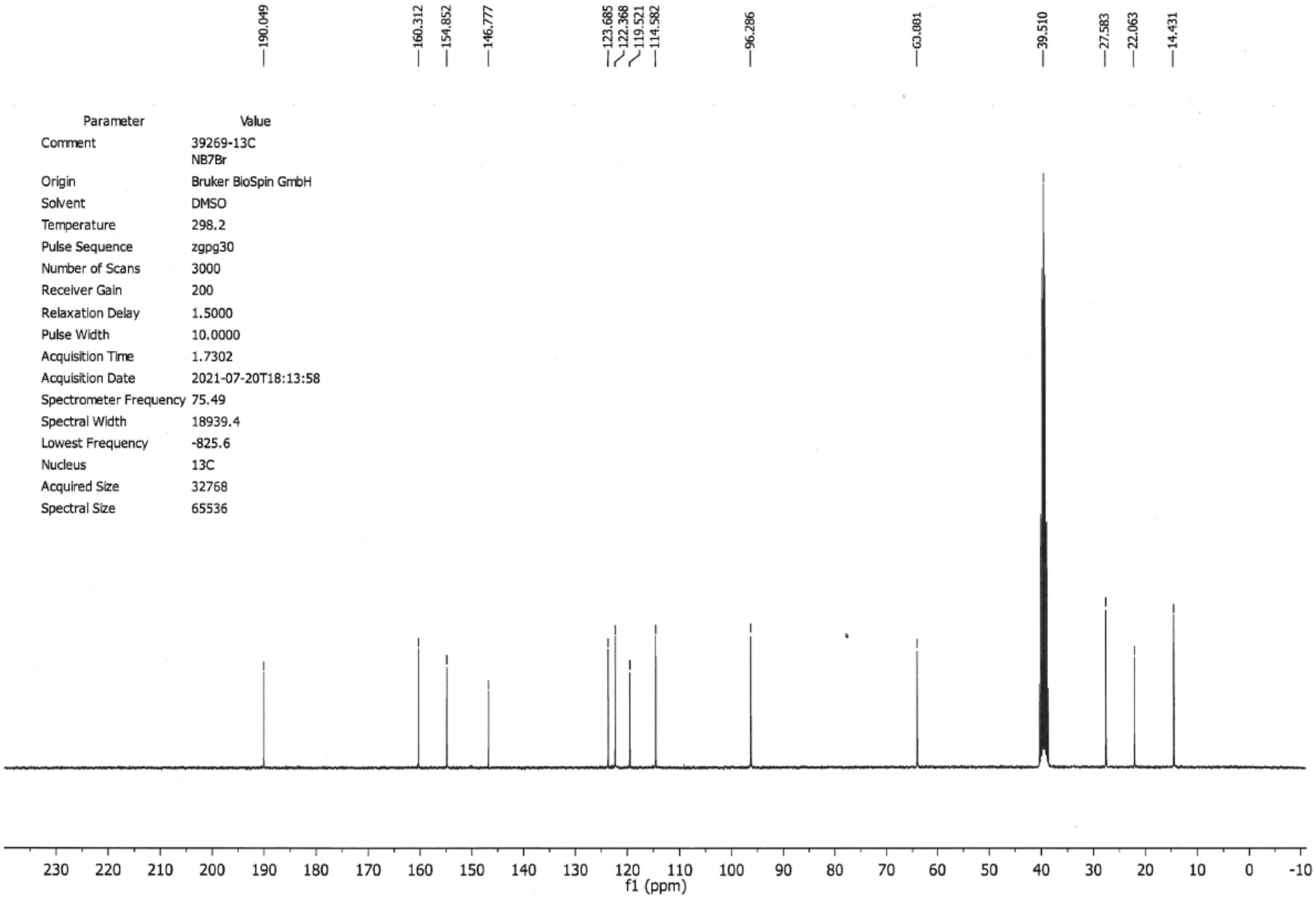

Supplement: Supplementary file 1 [file ijms-25-01999-s001.zip › ijms-2842594-supplementary.pdf]
